# New Hybrid Compounds Combining Fragments of Usnic Acid and Monoterpenoids for Effective Tyrosyl-DNA Phosphodiesterase 1 Inhibition

**DOI:** 10.3390/biom11070973

**Published:** 2021-07-01

**Authors:** Nadezhda S. Dyrkheeva, Aleksandr S. Filimonov, Olga A. Luzina, Alexandra L. Zakharenko, Ekaterina S. Ilina, Anastasia A. Malakhova, Sergey P. Medvedev, Jóhannes Reynisson, Konstantin P. Volcho, Suren M. Zakian, Nariman F. Salakhutdinov, Olga I. Lavrik

**Affiliations:** 1 Institute of Chemical Biology and Fundamental Medicine, Siberian Branch of the Russian Academy of Sciences, 630090 Novosibirsk, Russia; dyrkheeva.n.s@gmail.com (N.S.D.); a.zakharenko73@gmail.com (A.L.Z.); katya.plekhanova@gmail.com (E.S.I.); amal@bionet.nsc.ru (A.A.M.); medvedev@bionet.nsc.ru (S.P.M.); zakian@bionet.nsc.ru (S.M.Z.); 2 N.N. Vorozhtsov Novosibirsk Institute of Organic Chemistry, Siberian Branch of the Russian Academy of Sciences, 630090 Novosibirsk, Russia; alfil@nioch.nsc.ru (A.S.F.); luzina@nioch.nsc.ru (O.A.L.); anvar@nioch.nsc.ru (N.F.S.); 3 Federal Research Centre Institute of Cytology and Genetics, Siberian Branch of the Russian Academy of Sciences, 630090 Novosibirsk, Russia; 4 School of Pharmacy and Bioengineering, Keele University, Hornbeam Building, Staffordshire ST5 5BG, UK; j.reynisson@keele.ac.uk; 5 Department of Natural Sciences, Novosibirsk State University, 630090 Novosibirsk, Russia

**Keywords:** usnic acid, tyrosyl-DNA phosphodiesterase 1, TDP1 inhibitor, inhibiting activity, terpene, topotecan, synergy

## Abstract

Usnic acid (UA) is a secondary metabolite of lichens that exhibits a wide range of biological activities. Previously, we found that UA derivatives are effective inhibitors of tyrosyl-DNA phosphodiesterase 1 (TDP1). It can remove covalent complex DNA-topoisomerase 1 (TOP1) stabilized by the TOP1 inhibitor topotecan, neutralizing the effect of the drugs. TDP1 removes damage at the 3′ end of DNA caused by other anticancer agents. Thus, TDP1 is a promising therapeutic target for the development of drug combinations with topotecan, as well as other drugs for cancer treatment. Ten new UA enamino derivatives with variation in the terpene fragment and substituent of the UA backbone were synthesized and tested as TDP1 inhibitors. Four compounds, **11a**-**d**, had IC_50_ values in the 0.23–0.40 μM range. Molecular modelling showed that **11a**-**d**, with relatively short aliphatic chains, fit to the important binding domains. The intrinsic cytotoxicity of **11a**-**d** was tested on two human cell lines. The compounds had low cytotoxicity with CC_50_ ≥ 60 μM for both cell lines. **11a** and **11c** had high inhibition efficacy and low cytotoxicity, and they enhanced topotecan’s cytotoxicity in cancerous HeLa cells but reduced it in the non-cancerous HEK293A cells. This “protective” effect from topotecan on non-cancerous cells requires further investigation.

## 1. Introduction

**Figure 1:**
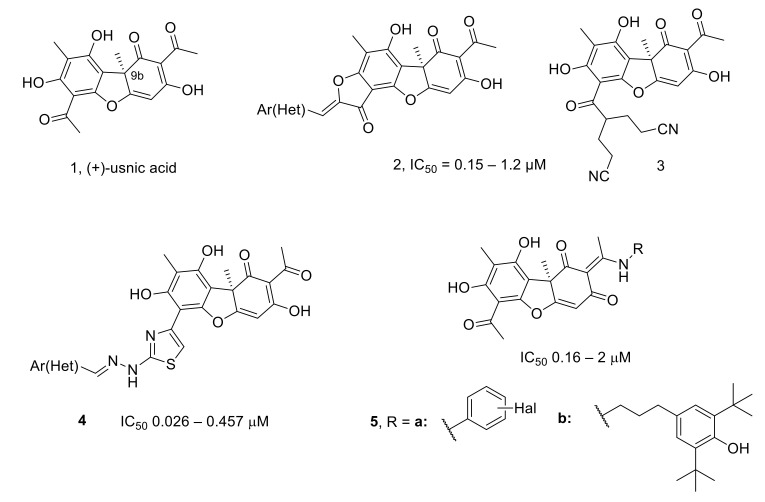
Structures of usnic acid (**1**) and its derivatives (**2**–**5**) that inhibit TDP1.

Usnic acid (UA, **1**, Figure 1) is a specific lichen metabolite. This bioactive substance is produced in large quantities by the lichen mycobiont, accounting for up to 8% of the dry weight of thalli. UA has antioxidant, antiviral, antibiotic, analgesic, antituberculosis, insecticidal, and anticancer activities [1,2], but with hepatotoxicity in high doses [3]. The main method of obtaining UA is the extraction from various genera of lichens such as *Cladonia* (Cladoniaceae), *Usnea* (Usneaceae), and others with organic solvents [4]. UA is a yellow crystalline substance with two enantiomeric forms differing in the orientation of the angular methyl group. Generally, only one of the enantiomers is isolated from each lichen species. The (+)-UA enantiomer is prevalent in nature and is commercially available [5].

Chemical modification of UA results in highly active derivatives against mycobacteria [6], viruses [7,8], and insects [9]; practically all the derivatives are less generally toxic than their parent [10,11].

Previously, we found that UA derivatives **2–5** (Figure 1) are effective inhibitors of tyrosyl-DNA phosphodiesterase 1 (TDP1) [12,13,14,15]. TDP1 plays a key role in DNA damage repair caused by antitumor drugs, such as the camptothecin derivatives [16,17]—topotecan (Tpc) and irinotecan. These drugs inhibit topoisomerase 1 (TOP1) that regulates the degree of local DNA torsion. A covalent DNA–TOP1 complex is formed [18], which is stabilized by the camptothecins, preventing the recovery of the DNA strand. Thus, DNA damage is preserved in the form of covalent adducts and single-strand breaks, leading to cell cycle arrest and death [18,19]. TDP1 can remove the DNA–TOP1 complex from the 3′ end, neutralizing the effect of TOP1 inhibitors [20]. Therefore, TDP1 can cause cellular resistance, and its suppression has the potential to enhance the therapeutic effect of the camptothecin analogs [21,22] Tpc and irinotecan [23], which are used in a range of tumors including lung cancer, colon cancer, cervical cancer, and ovarian cancer.

UA derivatives are highly effective TDP1 inhibitors (Figure 1) at low micromolar and nanomolar concentrations. We chemically modified UA to improve TDP1 inhibition and reduce general cytotoxicity [12,13,15,24]. Previously, we showed synergistic effects of UA derivatives with camptothecin or Tpc in vitro (compounds **3**–**5**, Figure 1) [12,13,14,15] and in vivo with Tpc (compounds **4** and **5b**, Figure 1) [12,25]. The hydrazinothiazole UA derivative with *para*-bromophenyl as the Ar ring (**4** in Figure 1) enhanced the antitumor and antimetastatic effect of Tpc in the mice Lewis lung carcinoma model [12]. Moreover, Tpc and **4** had an antitumor effect on Krebs 2 ascites tumors in mice separately; their combination considerably increased the antitumor potential [26]. Another UA derivative, an enamine with 3,5-di-*tert*-butyl-4-hydroxyphenyl substituent (**5b,** Figure 1), demonstrated a strong antimetastatic activity, under various administration schedules, by intravenous injection in mice with Lewis lung carcinoma [25]. Thus, UA-based TDP1 inhibitors can be considered promising for the development of combined antitumor therapy.

**Figure 2:**
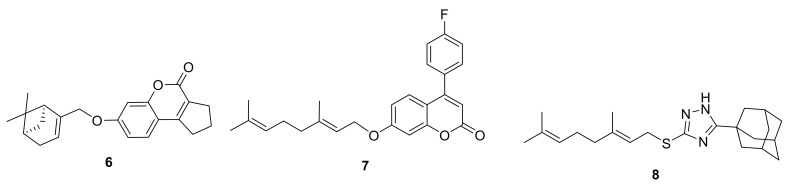
Structures of TDP1 inhibitors with monoterpenoid moieties.

Monoterpenoids are often used as starting molecules for the synthesis of new effective agents for the treatment of a wide range of diseases [27]; their use in drug design frequently reduces the toxicity of the resulting molecules. Various monoterpenoid-based TDP1 inhibitors (compounds **6**–**8**, Figure 2) are effective at submicromolar concentrations [28,29,30].

**Figure 3:**
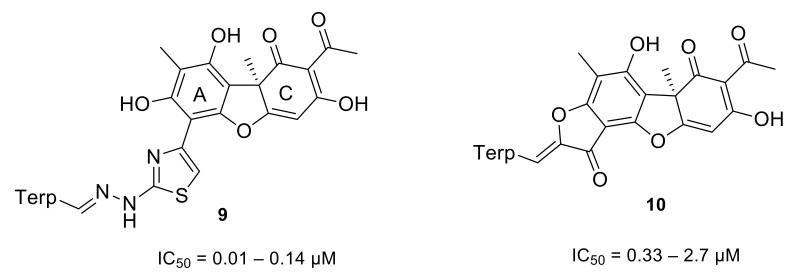
Structures of usnic acid and monoterpenoid conjugates that inhibit TDP1.

Previously, our team synthesized UA derivatives containing terpene fragments on ring A of the benzofuran backbone, hydrazinothiazole **9**, and aurone **10** (Figure 3) **[31,32]**. It was shown that substitution of UA with terpenes led to an increase in inhibitory activity (compound **9**) and to a decrease in the intrinsic cytotoxicity (compound **9** and **10**) compared to their aromatic (heteroaromatic) counterparts **2** and **4** (Figure 1).

UA enamine derivatives **5** are also effective [15]—they differ from the already mentioned aurones **2** and hydrazinothiazoles **4** by modification of the dibenzofuran backbone on the C ring. The IC_50_ values for these compounds ranged from 0.2 to 2 μM, and their distinctive feature is up to 12-fold sensitization of the MCF-7 (breast cancer) cells to Tpc [15].

The aim of this work was to synthesize enamino derivatives of UA with terpene fragments with variation both in the structure of the terpene fragment (linear, monocyclic, bicyclic) and to the location on the UA scaffold (directly or via a linker) and gauge their TDP1 inhibiting potential. Furthermore, cytotoxicity and sensitizing effects of cancer cell lines with Tpc were studied.

## 2. Materials and Methods

### 2.1. General Information

The analytical and spectral studies were conducted in the Chemical Service Center for the collective use of Siberian Branch of Russian Academy of Sciences.

The ^1^H and ^13^C NMR spectra in CDCl_3_ were recorded on a Bruker AVANCE III 400 spectrometer (Bruker Corporation, Germany; 400.13 and 100.61 MHz, respectively). The residual signals of the solvent were used as references (δ_H_ 7.24 and δ_C_ 76.9, respectively, for CDCl_3_). The mass spectra (70 eV) were recorded on a DFS Thermo Scientific high-resolution mass spectrometer (Thermo Fisher Scientific, Waltham, MA, USA). = Macherey-Nagel silica gel (63–200 μ) was used for the column chromatography. Thin-layer chromatography was performed on TLC Silica gel 60 plates (UV-254, Merck, Darmstadt, Germany).

All chemicals were used as described unless otherwise noted. Reagent-grade solvents were redistilled prior to use. Synthetic starting materials, reagents, and solvents were purchased from Sigma-Aldrich (St. Louis, MO, USA), Acros Organics (Geel, Belgium), and AlfaAesar (Heysham, UK). (*R*)-(+)-Usnic acid **1** (α_D_ +478 (c 0.1, CHCl_3_)) was isolated from a mixture of lichen genus *Usnea* by the Salakhutdinov et al. procedure [33]. Compound **12** was synthesized according to the literature [34]. Terpenoid amines were synthesized according to Suslov et al. [35].

The atom numbers in the compound are provided for the assignment of signals in the NMR spectra and are different from the numeration in the nomenclature name.

### 2.2. Chemistry

#### 2.2.1. General Procedure for the Synthesis of Enamine Compounds **11a**–**e**

**Figure 4:**
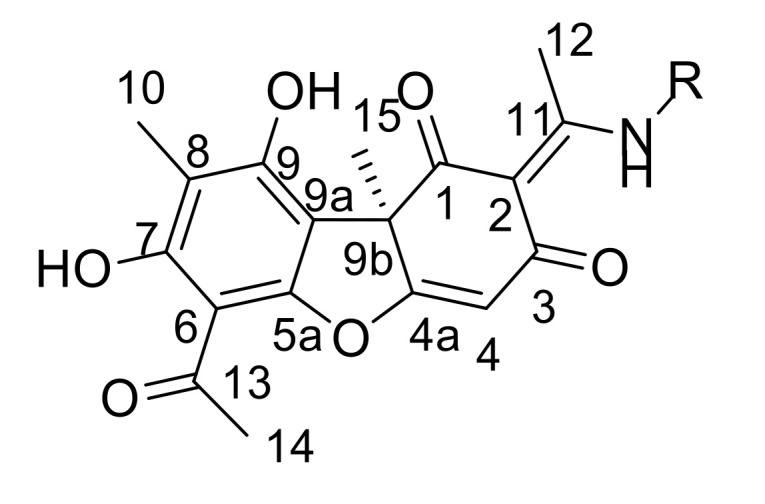
Structures of compounds 11**a**–**e** with atom numeration.

Usnic acid **1** (1 mmol), terpene amine (1.2 mmol), and NEt_3_ (0.2 mmol) were added to a dry flask. The mixture was dissolved in 10 mL of ethyl alcohol (95%) and boiled. After 2 h, the mixture was cooled, and aqueous hydrochloric acid was added to the mixture. The precipitate was filtered off, washed with water, and then air dried. The resulting solid was purified by chromatography over silica gel with CH_2_Cl_2_ to obtain desired products **11a**–**e** (Figure 4).

*(2R,4E)-10-Acetyl-4-(1-{[(3S)-3,7-dimethyloct-6-en-1-yl]amino}ethylidene)-11,13-dihydroxy-2,12-dimethyl-8-oxatricyclo[7.4.0.0^2,7^]trideca-1(13),6,9,11-tetraene-3,5-dione***11a**: Yellow amorphous powder with a 75% yield. NMR ^1^H: see Table 1. NMR ^13^C: see Table 2. HRMS: *m*/*z* 481.2457 [M]^+^ (calcd. for (C_28_H_35_O_6_N)^+^: 481.2459).

*(2R,4E)-10-Acetyl-4-(1-{[(2E)-3,7-dimethylocta-2,6-dien-1-yl]amino}ethylidene)-11,13-dihydroxy-2,12-dimethyl-8-oxatricyclo[7.4.0.0^2,7^]trideca-1(13),6,9,11-tetraene-3,5-dione***11b**: Yellow amorphous powder with a 68% yield. NMR ^1^H: see Table 1. NMR ^13^C: see Table 2. HRMS: *m*/*z* 479.2295 [M]^+^ (calcd. for (C_28_H_33_O_6_N)^+^: 479.2302).

*(2R,4E)-10-Acetyl-4-[1-({2-[(1R,5S)-6,6-dimethylbicyclo[3.1.1]hept-2-en-2-yl]ethyl}amino)ethylidene]-11,13-dihydroxy-2,12-dimethyl-8-oxatricyclo[7.4.0.0^2,7^]trideca-1(13),6,9,11-tetraene-3,5-dione* **11c**: Yellow amorphous powder with a 92% yield. NMR ^1^H: see Table 1. NMR ^13^C: see Table 2. HRMS: *m*/*z* 491.2307 [M]^+^ (calcd. for (C_29_H_33_O_6_N)^+^: 491.2302).

*(2R,4E)-10-Acetyl-4-[1-({[(1R,5S)-6,6-dimethylbicyclo[3.1.1]hept-2-en-2-yl]methyl}amino)ethylidene]-11,13-dihydroxy-2,12-dimethyl-8-oxatricyclo[7.4.0.0^2,7^]trideca-1(13),6,9,11-tetraene-3,5-dione* **11d**: Yellow amorphous powder with a 66% yield. NMR ^1^H: see Table 1. NMR ^13^C: see Table 2. HRMS: *m*/*z* 477.2146 [M]^+^ (calcd. for (C_28_H_31_O_6_N)^+^: 477.2144).

*(2R,4E)-10-Acetyl-11,13-dihydroxy-2,12-dimethyl-4-[1-({[(4S)-4-(prop-1-en-2-yl)cyclohex-1-en-1-yl]methyl}amino)ethylidene]-8-oxatricyclo[7.4.0.0^2,7^]trideca-1(13),6,9,11-tetraene-3,5-dione* **11e**: Yellow amorphous powder with a 33% yield. NMR ^1^H: see Table 1. NMR ^13^C: see Table 2. HRMS: *m*/*z* 477.2146 [M]^+^ (calcd. for (C24H18O8N432S2)^+^: 477.2151).

#### 2.2.2. NMR Spectra of Compounds **11a**–**e**

NMR ^1^H (**CDCl_3_**, δ): 1.7 (3H, s, H-15), 2.1 (3H, s, H-10), 2.6 (3H, s, H-12), 2.6 (3H, s, H-14), 5.8 (1H, s, H-4), 12.0 (1H, s, OH-9), 13.3 (2H, s and ss, OH-7 and NH). NMR ^13^C (**CDCl_3_**, δ): 7.4 (C-10), 20.9 (C-12), 31.1 (C-15), 31.2 (C-12), 56.6 (C-9b), 101.2 (C-9a), 102.1 (C-6), 102.4 (C-4), 105.0 (C-8), 107.8 (C-2), 155.8 (C-7), 158.2 (C-9), 163.3 (C-5a), 173.8 (C-11), 174.5 (C-4a), 189.4 (C-3), 198.0 (C-1), 200.6 (C-11).

#### 2.2.3. General Procedure for the Synthesis of Enamine Compounds **13a**–**e**

**Figure 5:**
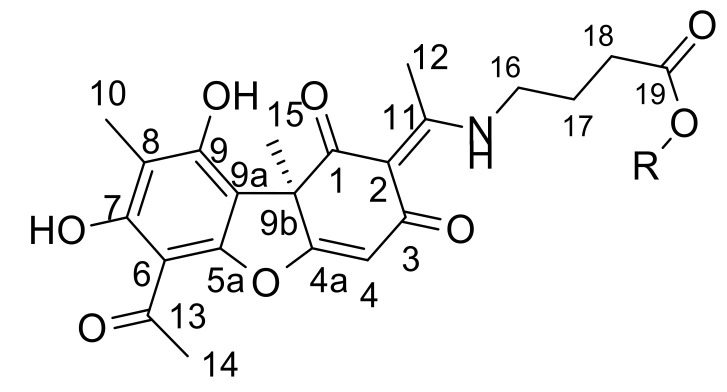
Structures of compounds 13a-e with atom numeration.

Usnic acid enamine **12** (1 mmol), terpene amine (1.3 mmol), 1-(3-dimethylaminopropyl)-3-ethylcarbodiimide hydrochloride (EDC) (1.45 mmol), and 4-dimethylaminopyridine (0.05 mmol) were dissolved in CH_2_Cl_2_ (10 mL) under argon. The mixture was stirred at 0 °C for 2 h. After that, mixture was stirred overnight at room temperature. The resulting mixture was washed with dilute HCl (0.1 M), aqueous NaHCO_3_ (0.1 M) and then brine. The combined organic extracts were dried over anhydrous Na_2_SO_4_, and the solvent was removed under reduced pressure. The resulting mixture was purified by chromatography over silica gel with CH_2_Cl_2_ to obtain desired products **13a**–**e** (Figure 5).

*(3R)-3,7-Dimethyloct-6-en-1-yl 4-({1-[(2R,4E)-10-acetyl-11,13-dihydroxy-2,12-dimethyl-3,5-dioxo-8-oxatricyclo[7.4.0.0^2,7^]trideca-1(13),6,9,11-tetraen-4-ylidene]ethyl}amino)butanoa*te **13a**: Yellow amorphous solid with a 53% yield. NMR ^1^H: see Table 3. NMR ^13^C: see Table 4. HRMS: *m*/*z* 567.2824 [M]^+^ (calcd. for (C_32_H_41_O_8_N)^+^: 567.2827).

*(2E)-3,7-Dimethylocta-2,6-dien-1-yl 4-({1-[(2R,4E)-10-acetyl-11,13-dihydroxy-2,12-dimethyl-3,5-dioxo-8-oxatricyclo[7.4.0.0^2,7^]trideca-1(13),6,9,11-tetraen-4-ylidene]ethyl}amino)butanoate* **13b**: Yellow amorphous solid with a 45% yield. NMR ^1^H: see Table 3. NMR ^13^C: see Table 4. HRMS: *m*/*z* 565.2667 [M]^+^ (calcd. for (C_32_H_39_O_8_N)^+^: 565.2670).

*2-[(1R,5S)-6,6-Dimethylbicyclo[3.1.1]hept-2-en-2-yl]ethyl 4-({1-[(2R,4E)-10-acetyl-11,13-dihydroxy-2,12-dimethyl-3,5-dioxo-8-oxatricyclo[7.4.0.0^2,7^]trideca-1(13),6,9,11-tetraen-4-ylidene]ethyl}amino)butanoate* **13c**: Yellow amorphous solid with a 69% yield. NMR ^1^H: see Table 3. NMR ^13^C: see Table 4. HRMS: *m*/*z* 577.2668 [M]^+^ (calcd. for (C_33_H_39_O_8_N)^+^: 577.2670).

*[(1R,5S)-6,6-Dimethylbicyclo[3.1.1]hept-2-en-2-yl]methyl 4-({1-[(2R,4E)-10-acetyl-11,13-dihydroxy-2,12-dimethyl-3,5-dioxo-8-oxatricyclo[7.4.0.0^2,7^]trideca-1(13),6,9,11-tetraen-4-ylidene]ethyl}amino)butanoate* **13d**: Yellow amorphous solid with a 53% yield. NMR ^1^H: see Table 3. NMR ^13^C: see Table 4. HRMS: *m*/*z* 563.517 [M]^+^ (calcd. for (C_32_H_37_O_8_N)^+^: 563.2514).

*[(4S)-4-(Prop-1-en-2-yl)cyclohex-1-en-1-yl]methyl 4-({1-[(2R,4E)-10-acetyl-11,13-dihydroxy-2,12-dimethyl-3,5-dioxo-8-oxatricyclo[7.4.0.0^2,7^]trideca-1(13),6,9,11-tetraen-4-ylidene]ethyl}amino)butanoate* **13e**: Yellow amorphous powder with a 68% yield. NMR ^1^H: see Table 3. NMR ^13^C: see Table 4. HRMS: *m*/*z* 563.2508 [M]^+^ (calcd. for (C_32_H_39_O_8_N)^+^: 563.2514).

#### 2.2.4. NMR Spectra of Compounds **13a**–**e**

NMR ^1^H (**CDCl_3_**, δ): 1.7 (3H, s, H-15), 2.1 (3H, s, H-10), 2.1 (2H, p, J = 6.9 Hz), 2.6 (3H, s, H-12), 2.6 (3H, s, H-14), 2.45 (2H, t, J = 6.9 Hz), 3.52 (2H, m, H-16), 5.8 (1H, s, H-4), 12.0 (1H, s, OH-9), 13.3 (2H, s and ss, OH-7 and NH). NMR ^13^C (**CDCl_3_**, δ): 7.4 (C-10), 20.9 (C-12), 24.2 (C-17), 30.8 (C-18), 31.1 (C-15), 31.2 (C-12), 42.9 (C-16), 56.6 (C-9b), 101.2 (C-9a), 102.1 (C-6), 102.4 (C-4), 105.0 (C-8), 107.8 (C-2), 155.8 (C-7), 158.2 (C-9), 163.3 (C-5a), 172.1 (C-19), 173.8 (C-11), 174.5 (C-4a), 189.4 (C-3), 198.0 (C-1), 200.6 (C-11).

### 2.3. Biology

#### 2.3.1. Real-Time Detection of TDP1 Activity

The oligonucleotide biosensor 5′-FAM-AAC GTC AGG GTC TTC C- BHQ1-3′ was used for TDP1 enzyme activity real-time fluorescence detection [36], that is, a 16-mer single-stranded oligonucleotide with a 5′-fluorophore (FAM), and a 3′-quencher (BHQ1). Recombinant protein TDP1 was expressed in *E. coli* (pET 16B) plasmid containing TDP1 cDNA, kindly provided by Dr. K.W. Caldecott (University of Sussex, United Kingdom) and isolated as described [37]. The final volume of 200 μL of reaction mixture contained TDP1 reaction buffer (50 mM Tris-HCl (pH 8.0), 50 mM NaCl, 7 mM β-mercaptoethanol), 50 nM oligonucleotide substrate, and varied concentrations of the tested compounds. Purified TDP1 was added in a final concentration of 1.5 nM.

The TDP1 reaction mixtures were incubated at a constant temperature of 26 °C in a POLARstar OPTIMA fluorimeter (BMG LABTECH, GmbH). Fluorescence intensity was measured (Ex. 485/Em. 520 nm) every 1 min for 10 min. The efficiency of TDP1 inhibition was evaluated by comparing the rate of increase in fluorescence of biosensor in the presence of compound to that of DMSO (1.5%) in control wells. IC_50_ values were determined using an 11-point concentration response curve by MARS Data Analysis 2.0 (BMG LABTECH), and the slope during the linear phase was calculated. The IC_50_ measurements were carried out in at least three independent experiments.

#### 2.3.2. TDP1 Activity by gel-Based Assay

Oligonucleotide 5′- FAM-AAC GTC AGG GTC TTC C-tyrosine-3′ was used for the indication of TDP1 enzyme activity in polyacrylamide gel. The oligonucleotide containing a natural phosphotyrosine adduct at the 3′-end is a substrate for TDP1 [38]. The reaction mixture was conducted in the reaction buffer (50 mM Tris-HCl, 50 mM NaCl, 7 mM β-mercaptoethanol), 50 nM oligonucleotide, 5 nM purified TDP1, or 1 μg of cell extract in a final volume 20 μL at 37 °C for 20 min. The reaction products were separated by electrophoresis in a 20% denaturing polyacrylamide gel with 7 M urea. A Typhoon FLA 9500 phosphorimager (GE Healthcare, Uppsala, Sweden) was used for gel scanning and imaging, and the data were analyzed with QuantityOne 4.6.7 software.

#### 2.3.3. TDP1 Knockout HEK293A Clones

TDP1 knockout HEK293A clones were obtained in the same way as the previously described TDP1 knockout HEK293FT clones [39]. Briefly, two 20-nt protospacer sequences flanking the first protein coding exon of human TDP1 gene were selected using https://www.benchling.com/ (accessed date 1 November 2020). Oligonucleotides were cloned in plasmid pSpCas9(BB)-2A-GFP (PX458). pSpCas9(BB)-2A-GFP (PX458) was a gift from Feng Zhang (Addgene plasmid # 48138; http://n2t.net/addgene:48138; RRID: Addgene_48138). HEK293A cells were transfected with plasmids pX458-TDP1-gRNA1 and pX458-TDP1-gRNA2 (0.25 µg of each) using Lipofectamine 3000 Reagent (Thermofisher Scientific, Waltham, MA, USA). Growth medium contained DMEM/F12 (Thermofisher Scientific, Waltham, MA, USA) 1:1, 10% FBS (Thermofisher Scientific, Waltham, MA, USA), 100 U/mL penicillin–streptomycin (Thermofisher Scientific, Waltham, MA, USA), and 1 × GlutaMAX (Thermofisher Scientific, Waltham, MA, USA). Forty-eight hours after transfection, the GFP-positive cell population was enriched by cell sorting using BD FACSAria III Cell Sorter (BD Biosciences, Franklin Lakes, NJ, USA). Single cell clones grew for two weeks, and genome DNA was analyzed on CRISPR/Cas9-mediated deletions in TDP1 gene by PCR amplification of target region with primers: TDP1-scF 5′-TCAGGAAGGCGATTATGGGAG-3′ and TDP1-scR 5′-TTGATGTGGAGGGCTCCAG-3′. As a result, three HEK293A clones were found, which contained only alleles with deletions (C6, G6, and F7). PCR products obtained from genomic DNA of these clones were gel purified and cloned in the pGEM-T Easy vector (Promega, Madison, WI, USA). Ten independent plasmid clones were isolated and Sanger sequenced using M13 universal primers.

#### 2.3.4. Cell Culture Cytotoxicity Assay

Cytotoxicity of the compounds was examined against human cell lines HEK293A (human embryonic kidney)—WT and TDP1-deficient (Tdp1-/-), and HeLa (cervical cancer) using an EZ4U colorimetric tests (Biomedica, Vienna, Austria) or xCELLigence DP Real-Time Cell Analyzer (ACEA Biosciences, Santa Clara, CA, USA). Cell lines were obtained from the Russian Cell Culture Collection (RCCC) Institute of Cytology RAS (St. Petersburg, Russia). The cells were grown in DMEM/F12 medium (Thermo Fisher Scientific, Waltham, MA, USA), with 1× GlutaMAX (Thermo Fisher Scientific, Waltham, MA, USA), 50 IU/mL penicillin, and 50 μg/mL streptomycin (Thermo Fisher Scientific, Waltham, MA, USA), and in the presence of 10% fetal bovine serum (Biolot, Saint-Petersburg, Russia) in 5% CO_2_ atmosphere. Control cells were grown in the presence of 1% DMSO where indicated. After formation of a 30–50% monolayer, tested compounds were added to the medium, and the cell culture was monitored for 3 days. The compound concentration that caused 50% cell growth inhibition (CC_50_) was determined using OriginPro 8.6.0 software. The measurements were carried out in three parallel experiments.

### 2.4. Modelling and Screening

The compounds were docked against the crystal structure of TDP1 (PDB ID: 6W7K, resolution 1.70 Å) [40], which was obtained from the Protein Data Bank (PDB) [41,42]. The Scigress version FJ 2.6 program [43] was used to prepare the crystal structure for docking, i.e., the hydrogen atoms were added and the co-crystallized ligand 4-[(2-phenylimidazo[1,2-*a*]pyridin-3-yl)amino]benzene-1,2-dicarboxylic acid (TG7) was removed. The Scigress software suite was also used to build the inhibitors and the MM2 [44] force field was applied to identify the global minimum using the CONFLEX method [45], followed by structural optimization. The docking center for the catalytic pocket was defined as the position of the nitrogen atom in the amine group of TG7 (x = 10.622, y = −3.446, z = −99.112) with 10 Å radius. The center for the allosteric site was defined as the oxygen atom in the carboxylic group pointing into the pocket (x = 18.656, y = −0.525, z = −98.790). Fifty docking runs were allowed for each ligand with default search efficiency (100%). The basic amino acids lysine and arginine were defined as protonated. Furthermore, aspartic and glutamic acids were assumed to be deprotonated. The GoldScore (GS) [46] and ChemScore (CS) [47,48] ChemPLP (Piecewise Linear Potential) [49] and ASP (Astex Statistical Potential) [50] scoring functions were implemented to predict the binding modes and relative energies of the ligands using the GOLD v5.4.1 software suite.

The QikProp 6.2 [51] software package was used to calculate the molecular descriptors of the molecules. The reliability of QikProp was established for the calculated descriptors [52]. The Known Drug Indexes (KDI) were calculated from the molecular descriptors as described by Eurtivong and Reynisson [53]. For application in Excel, columns for each property were created and the following equations were used to derive the KDI numbers for each descriptor: KDI MW: = EXP(-((MW-371.76)^2)/((2x112.76)^2)), KDI Log P: = EXP(-((LogP-2.82)^2)/((2x2.21)^2)), KDI HD: = EXP(-((HD-1.88)^2)/((2x1.7)^2)), KDI HA: = EXP(-((HA-5.72)^2)/((2x2.86)^2)), KDI RB = EXP(-((RB-4.44)^2)/((2x3.55)^2)), and KDI PSA: = EXP(-((PSA-79.4)^2)/((2x54.16)^2)). These equations could simply be copied into Excel and the descriptor name (e.g., MW) substituted with the value in the relevant column. To derive KDI_2A_, we used the following equation: = (KDI MW + KDI LogP + KDI HD + KDI HA + KDI RB + KDI PSA), and for KDI_2B_: = (KDI MW × KDI LogP × KDI HD × KDI HA × KDI RB × KDI PSA).

## 3. Results

### 3.1. Chemistry

The target UA enamine derivatives **11a**–**e** with terpene substituents were synthesized by reacting usnic acid and the corresponding terpeneamines **14a**–**e**. The UA derivatives, with terpene moiety connected to UA via γ-aminobutyric acid (GABA), were synthesized in two steps. At the first stage, the UA enamine derivative **12** with GABA substituent was synthesized via reaction of usnic acid with GABA. At the second stage, compound **12** reacted with terpeoles to produce the target UA derivatives **13a**–**e**.

#### 3.1.1. Synthesis of Usnic Acid Enamine Derivatives **11a**–**e**

**Scheme 1:**
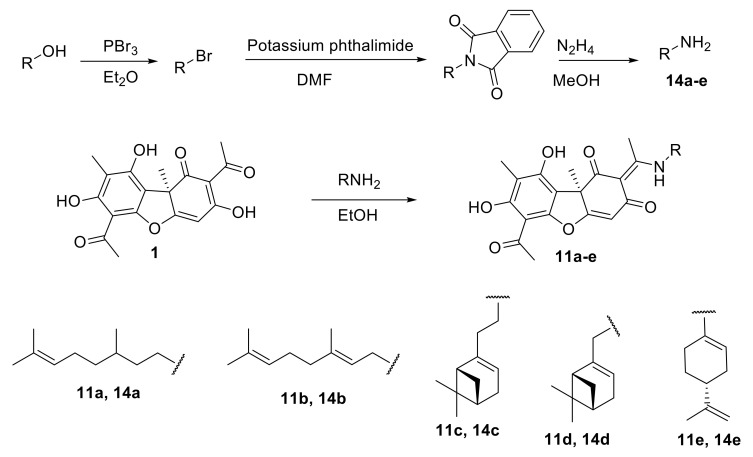
Synthesis of compounds **11a**–**e**.

At first, we synthesized corresponding amines from commercially available monoterpene alcohols. A set of monoterpenoid amines of different structural types was synthesized by the Gabriel method [54]. Citronellylamine **14a**, geranylamine **14b**, nopylamine **14c**, myrtenylamine **14d**, and perillamine **14e** were obtained by bromination of the corresponding alcohols followed by reaction with potassium phthalimide and reduction with hydrazine to the target amine (Scheme 1). Reaction of (+)-UA **1** with terpenoids **14a–e** with amino group resulted in corresponding conjugates with the enamino structure **11a–e**. Thus, five new compounds, derivatives of UA with mono-, bi-, and acyclic terpene fragments, were obtained.

#### 3.1.2. Synthesis of Usnic Acid Enamine Derivatives with an Ether Bond **13a**–**e**

**Scheme 2:**
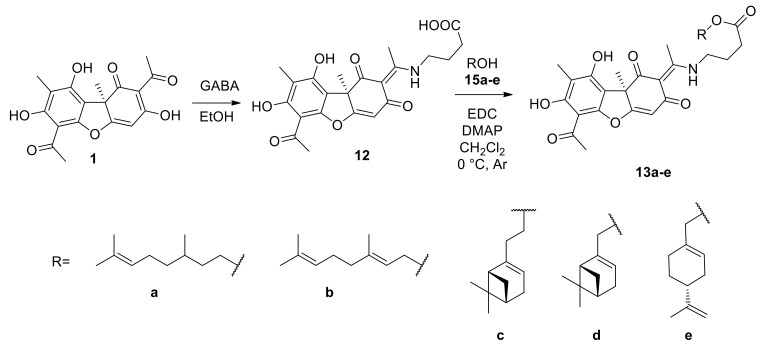
Synthesis of compounds **13a**–**e**.

The derivative **12** was synthesized according to a previously developed technique by reaction of (+)-UA **1** with γ-aminobutyric acid (GABA) (Scheme 2) [34]. GABA was chosen as an amino acid linker to bind the two pharmacophores on the basis of its availability and simplification of product characterization as it does not contain chiral centers.

For synthesis of compounds **13a–e**, commercially available citronellol, geraniol, and nopol were used. Terpene alcohols **15d** and **15e** were obtained by reduction of myrtenal and perillyl aldehyde with sodium borohydride in methanol. Reaction of these alcohols with **12** led to formation ester derivatives **13a–e** with the terpene fragment at the C ring of UA (Scheme 2). The reaction was carried out in dry methylene at 0 °C and in an inert atmosphere using EDC as a condensing agent and dimethylaminopyridine (DMAP) as a catalyst.

### 3.2. Biology

#### 3.2.1. Real-Time Detection of TDP1 Activity

We tested the 10 enamine derivatives of (+)-UA for their TDP1 inhibitory properties by deriving their IC_50_ values (the inhibitor concentration required to suppress the activity of enzyme by 50%) using a real-time fluorescent test according to the method we developed [36]. Enamine derivatives in which the terpene fragment is connected to the (+)-UA scaffold via a linker with an ester bond (compounds **13a–e**) did not show pronounced TDP1 inhibition (IC_50_ ≥ 10 μM, Table 5). At the same time, terpene–UA conjugates obtained by direct condensation of UA with primary terpenoid amines (compounds **11a–d**) showed inhibitory activity at submicromolar concentrations. IC_50_ values for the most effective inhibitors (**11a**, **11b**, **11c**, **11d**) varied in a narrow range of 0.23–0.40 μM. Thus, the inhibitory activity only slightly depends on the structure of the terpene part, excluding **11e** with monocyclic *para*-menthane fragment.

Thus, compounds containing an acyclic (**11a**, **11b**) or bicyclic (**11c**, **11d**) terpene fragment proved to be potent inhibitors of TDP1, and they were selected for cytotoxicity studies.

#### 3.2.2. Cell Culture Cytotoxicity Assay

**Figure 6:**
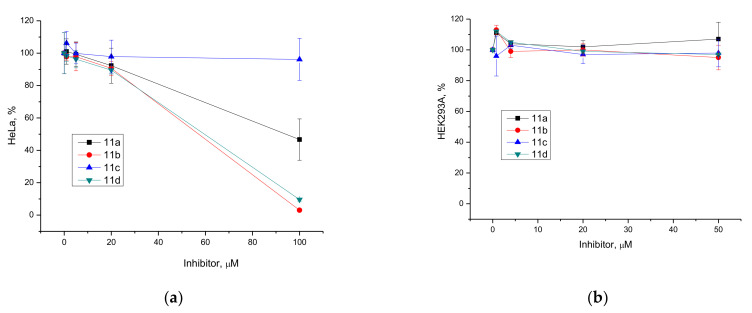
The TDP1 inhibitors’ intrinsic cytotoxicity on HeLa (**a**) and HEK293A (**b**) cells—dose-dependent action.

The intrinsic cytotoxicity of the four lead compounds (**11a–d**) was examined using a colorimetric test (Figure 6). The derivatives exhibited low cytotoxicity (CC_50_ ≥ 60 μM) for both HeLa and HEK293A cells, and the dose response graphs are shown in Figure 6 and Figure S1.

As can be seen in Figure 6, both **11b** and **11d** were more cytotoxic than **11a** and **11c**; the former completely suppressed cell growth at 100 μM. **11a** and **11c**, with acyclic terpene fragment, are effective TDP1 inhibitors and non-cytotoxic for HeLa and HEK293A cells. Low intrinsic cytotoxicity of TDP1 inhibitors is desired for their use as sensitizers with, e.g., Tpc.

#### 3.2.3. Potentiation with Topotecan

**Figure 7:**
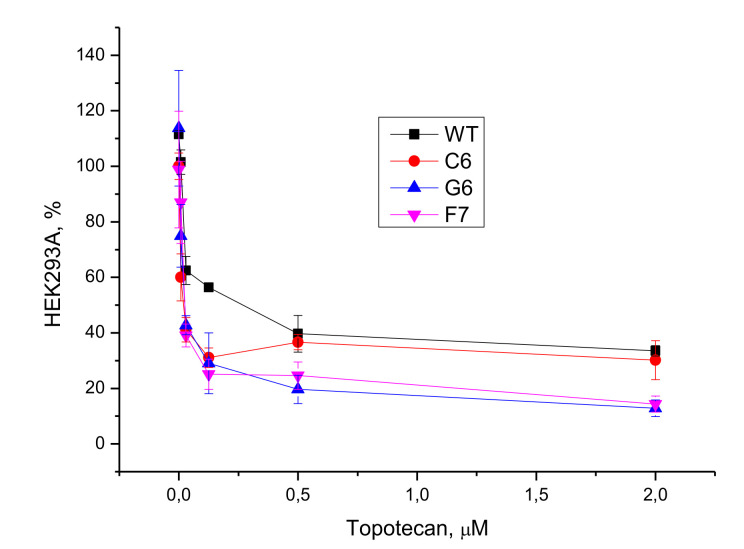
Topotecan (Tpc) cytotoxicity in HEK293A WT and TDP1 -/- knockout cell lines C6, G6 and F7—dose-dependent action of Tpc by colorimetric test.

In our previous work [39], the cytotoxic effect of Tpc and the TDP1 inhibitors, monoterpene 3-carene derivatives, was established; both were measured separately and jointly using a panel of HEK293FT TDP1 knockout isogenic clones. It was shown that HEK293FT TDP1-/- cells were more sensitive to Tpc compared to WT (wild-type) cells. The data on HEK293FT mutants were of low reproducibility due to the weak cell adherence; it was therefore decided to change the cell line to HEK293A. In this work, we created a new panel of TDP1-/- HEK293A cells using the CRISPR/Cas9 and polymerase chain reaction (PCR) screening of cell clones (Figure S21). Three clones contained deletions in the first protein coding exon of TDP1 gene: clone C6 Δ196bp/Δ197, clone G6 Δ197bp/Δ197bp, and clone F7 Δ198bp/Δ196bp (Figures S21 and S22A). The clones C6, G6, and F7 were screened by a biochemical assay for 3′-phosphotyrosyl cleavage to identify TDP1 activity in the cell extract (Figure S22B). No cleavage activity was detected in the clones’ C6, G6, and F7 cell extracts in contrast to control WT and purified TDP1. We treated TDP1-proficient (WT) and TDP1-deficient (TDP1-/-) cells with increasing concentrations of Tpc. The results for the three clones are presented in Figure 7. The same effect was seen as for the HEK293FT cell line [39], and TDP1-/- cells of the HEK293A cell line were more sensitive to Tpc compared to WT (Figure 7).

TDP1 can work against the action of Tpc, cleaving the TOP1–DNA stalled complex. Next, we checked the cytotoxic effect of the combination of Tpc and **11a–d** as compared to Tpc in the HeLa cancer cells and non-cancerous HEK293A WT and TDP1-/- cells.

**Figure 8:**
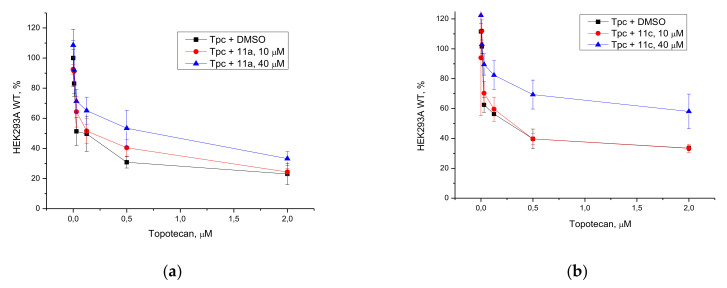
Topotecan (Tpc) cytotoxicity on HEK293A WT cells—dose-dependent action of Tpc in combination with **11a** (**a**) or **11c** (**b**).

Surprisingly, when we added combination of Tpc and the inhibitors (**11a** and **11c**) to the HEK293A cells, a stimulation of cell growth was observed as compared to Tpc for both WT (Figure 8) and Tdp1-/- cells (Figure S23). There was no difference between WT and the mutant cells.

**Figure 9:**
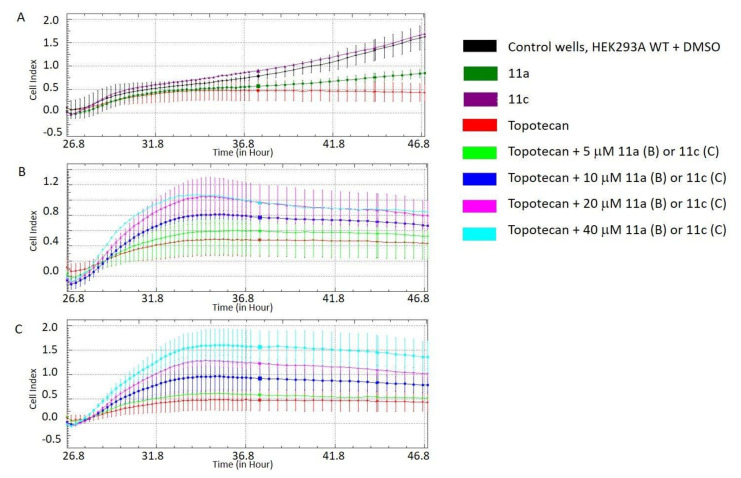
Compounds **11a** and **11c** stimulated cell growth of HEK293A WT cells with Tpc, as compared to Tpc, using the impedance-based real-time assay. (**A**). Cell growth of HEK293A cells in the control wells without compounds added (black), with 50 nM Tpc (red), 40 µM **11a** (dark green), or 40 µ M **11c** (violet). (**B**,**C**) Cell growth of HEK293A cells with 50 nM Tpc only (red) and in combination with 5 µM (green), 10 µM (dark blue), 20 µM (pink), or 40 µM (blue) **11a** (**B**) or **11c** (**C**).

This stimulation was also confirmed for HEK293A WT cells using the xCELLigence DP Real-Time Cell Analyzer impedance-based assay (Figure 9). As seen previously, **11c** was non-toxic and **11a** was modestly toxic for HEK293A cells (Figure 9A, dark green and violet). The stimulation effect depends on the concentration of compounds **11a** and **11c** (Figure 9B,C). Thus, both **11a** and **11c** stimulated cell growth of non-cancerous HEK293A cells when dosed with Tpc.

**Figure 10:**
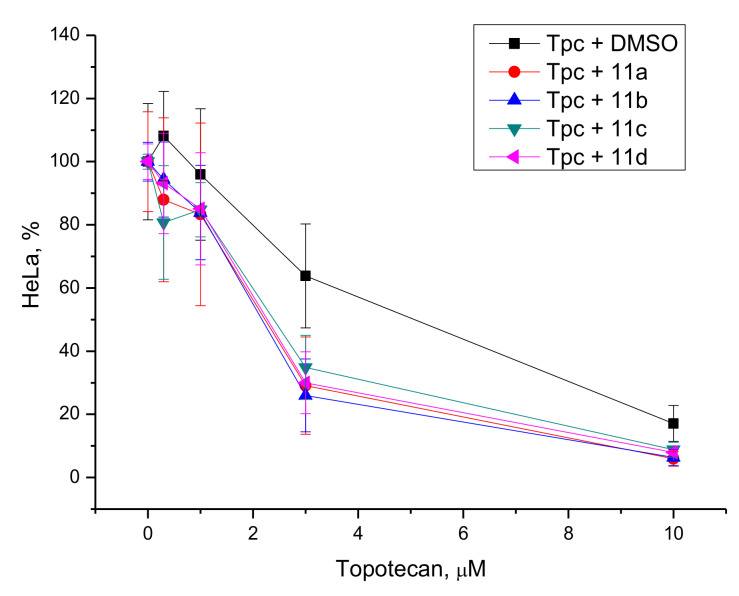
Dose-dependent action of the Tpc with **11a**, **11b**, **11c**, and **11d** (5 µM) on HeLa cells’ viability.

In cancer HeLa cells, all four TDP1 inhibitors showed promising synergy in conjunction with Tpc (Figure 10). The combination index (CI) values were determined for different concentrations of Tpc and the enamines **11a–d** at 2 and 5 μM. They were predominantly in the range 0.5–0.8, indicating a synergistic effect between Tpc and the UA derivatives. The CI values were calculated with the CompuSyn version 1.0 software. The CI plot at different fraction-affected (Fa) was determined for Tpc (0.3, 0.5, 1, 2, and 4 μM) and the Tdp1 inhibitors (2 and 5 μM). The CI values for most of the combinations corresponded to a synergistic effect (CI < 1, see Figure S24 in the Supplementary Materials).

Both **11a** and **11c** showed synergistic effect with Tpc reducing viability in the HeLa cell line, but stimulated cell growth of non-cancerous HEK293A cells.

### 3.3. Modelling and Screening

The 10 UA enamine derivatives were docked into the binding site of the TDP1 structure (PDB ID: 6W7K, resolution 1.70 Å) [40]. The co-crystalized ligand (TG7) was removed and re-docked into the binding site to test the robustness of the scoring functions GoldScore (GS) [46] and ChemScore (CS) [47,48] ChemPLP (Piecewise Linear Potential) [49] and ASP (Astex Statistical Potential) [50] in the GOLD (v2020.2.0) docking algorithm (the validation of the quality of the docking algorithm is given in Table S1). 

It has been suggested, on the basis of molecular dynamics simulations, that the UA enamines occupy an allosteric site next to the catalytic pocket, thus reducing TDP1 activity [15]. This pocket is made up of the Tyr204, Cys205, Asp230, Lys231, Leu255, Ala258, Phe259, and Thr261 amino acid residues, which can form hydrophobic contacts and hydrogen bonding interactions with small molecular ligands. The ligands were docked to this site, and in most cases, the GOLD algorithm directed the ligands into the catalytic pocket, predicting the same, or similar, conformations as seen previously. Simulating DNA occupancy of the catalytic pocket by leaving the co-crystallized inhibitor in it directed the docking to the allosteric site. The scores generated were realistic (see Table S2); correlations with the measured IC_50_ values were seen, but the slope of the fitted line was positive, i.e., the higher the scores, the poorer the inhibition. Moreover, the averages of the inactive ligands (>10 µM) were higher than for those with good activity.

**Figure 11:**
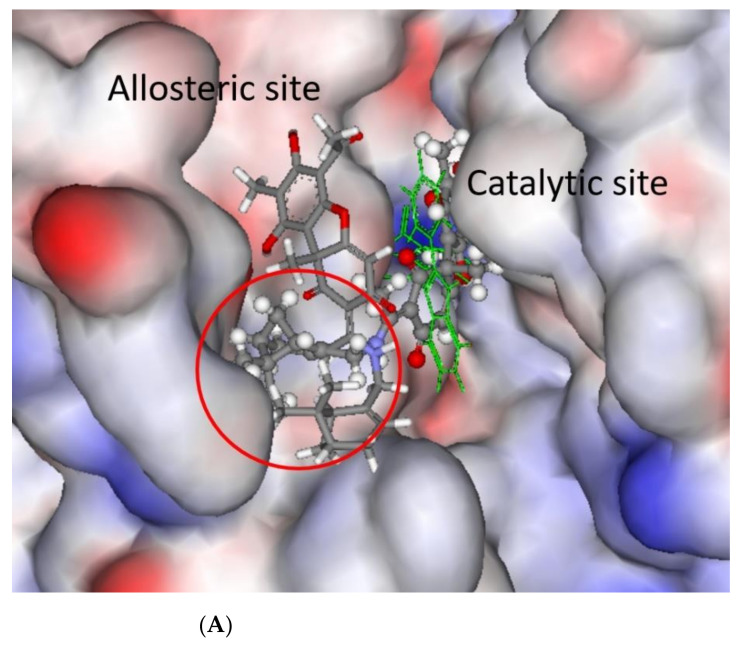

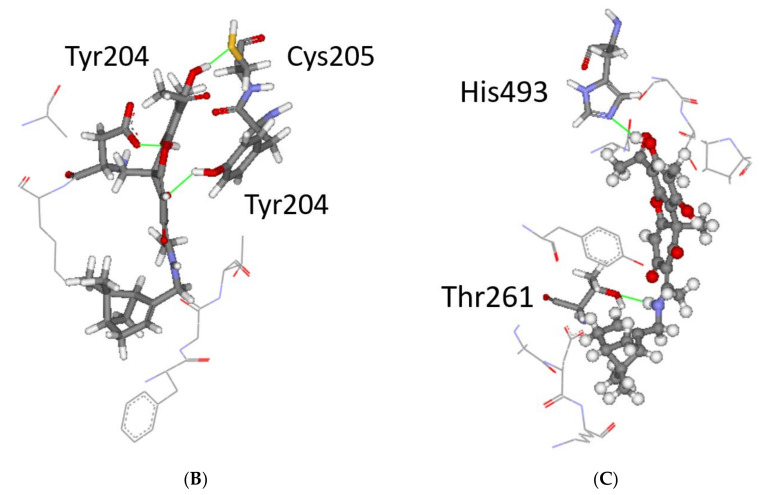
The docked poses of **11d** in both potential binding sites of TDP1 as predicted by the ASP scoring function. (**A**) The allosteric site is to the left-hand side, and the ligand’s predicted conformation is shown in stick format. The catalytic pocket is to the right-hand side, and the ligand is depicted as balls and sticks, reaching into the allosteric pocket with its aliphatic bicyclic ring (see circled area). The co-crystalized ligand is shown (green lines) buried in the catalytic pocket. The protein surface is rendered; blue depicts regions with a partial positive charge on the surface, red depicts regions with a partial negative charge, and gray shows neutral areas. (**B**) Allosteric site: The predicted configuration is shown in the stick format as well as Tyr204, Cys205, and Asp230, which formed hydrogen bonds (green lines) to the ligand. Adjacent amino acid residues buttressing the ligand are shown in the line format (Lys231, Ala234, Phe259, Gly260, Thr261); the hydrogen atoms are not shown for clarity. (**C**) Catalytic pocket: The predicted configuration depicted as balls and sticks with hydrogen bonding amino acid residues Thr261 and His493. The adjacent amino acids are Tyr204, Gly229, Asp230, Lys231, Phe259, Gly260, Ser399, Ser400, Pro461, and Asn516.

It can therefore be concluded that both scenarios were possible, and that potentially both took place in the assay and biological systems. This question can be answered with X-ray crystallography. The predicted binding of **11d** is given in Figure 11 for both binding sites.

As can be seen in Figure 11A, the 6,6-dimethylnopin-2-ene ring occupied both binding sites, i.e., the UA scaffold was predicted to be buried in the catalytic pocket whereas the aliphatic ring stretched into the allosteric site. In both scenarios, plausible binding was observed, with hydrogen bonding anchoring the ligand in place as well as scaffolding of lipophilic amino acid residues buttressing the bound ligand. The predicted conformation in the allosteric site was more exposed to the aqueous phase as compared to the binding in the catalytic pocket.

When the predicted conformations were analyzed, then the derivatives with long aliphatic chains (**13a**–**e**) did not fit into the binding domain; the aliphatic chain was too long to allow for the binding of the UA scaffold into the catalytic pocket and for the aliphatic rings, or saturated chains, to fit in the allosteric binding pocket, as shown in Figure 11A, C for **11d**. The rest of the ligands (**11a**, **11b**, and **11c**) had relatively short chains and predominantly were predicted, by the scoring functions used, to adopt the same conformation as **11d** in the catalytic pocket. Interestingly, compound **11e** did not have inhibitory activity (IC_50_ > 10 µM), even though it is a close analogue of **11d**; when the binding of **11e** was analyzed, only CS predicted a clear bind mode with the UA moiety in the catalytic pocket and the aliphatic ring in the allosteric binding site. Ample hydrogen bonding was predicted for the UA to various amino acid residues in the catalytic pocket, but no hydrogen bonding was seen in the allosteric site. This means that the monocyclic ring did not fit well into the allosteric site and did not facilitate hydrogen bonding anchoring it in place, in comparison with **11d** that formed a hydrogen bond with Thr261 in the allosteric site. From these observations, a plausible binding mode is presented in Figure 11A, C for the catalytic pocket, which agrees with the structure–activity relationship.

### 3.4. Chemical Space

The calculated molecular descriptors MW (molecular weight), log P (water-octanol partition coefficient), HD (hydrogen bond donors), HA (hydrogen bond acceptors), PSA (polar surface area), and RB (rotatable bonds)) are given in Table S3. The values of the molecular descriptors lay within drug-like chemical space and Known Drug Space (KDS), except for HD, which was in lead-like space. HA and RB were in drug-like chemical space, whereas the rest of the descriptors straddled drug-like space and KDS (for the definition of lead-like, drug-like, and KDSregions, see [55] and Table S4). Interestingly, when the descriptor values were correlated with their IC_50_ counterparts, albeit only four, very good correlation was seen for RB (R^2^—0.799); Log P (R^2^—0.849); and, to a lesser extent, PSA (R^2^—0.332). When one outlier (**11c**) was removed from MW, then an almost perfect correlation of R^2^—0.999 was derived (see Figures S25–S28 in the Supplementary Materials). In all four cases, smaller descriptor values led to improved inhibition. In addition, when the averages were calculated for the active and inactive (IC_50_ >10 µM) derivatives, considerably smaller values were seen for all the molecular descriptors used, except HD for the active derivatives (see Table S2). This led to the conclusion that smaller compounds are favorable for the UA enamines. Correlations between the molecular descriptors and their corresponding binding efficacies to TDP1 have been previously reported for deoxycholic acid derivatives, MW of R^2^—0.452 and of 0.316 for RB, [56] as well as for sultone-fused berberine derivatives for MW (R^2^—0.735) and log P (R^2^—0.613) [57].

The Known Drug Indexes (KDIs) for the ligands were calculated to gauge the balance of the molecular descriptors (MW, log P, HD, HA, PSA, and RB). This method is based on the analysis of drugs in clinical use, i.e., the statistical distribution of each descriptor is fitted to a Gaussian function and normalized to 1, resulting in a weighted index. Both the summation of the indexes (KDI_2a_) and multiplication (KDI_2b_) methods were used [53], as shown for KDI_2a_ in Equation (1) and for KDI_2b_ in Equation (2); the numerical results are given in Table S3 in the Supplementary Materials section.

KDI_2a_ = I_MW_ + I_log P_ + I_HD_+ I_HA_ + I_RB_ + I_PSA_ 2) (1)

KDI_2b_ = I_MW_ × I_log P_ × I_HD_× I_HA_ × I_RB_ × I_PSA_ 3) (2)

The KDI_2a_ values for the ligands ranged from 3.43 to 5.27, with a theoretical maximum of 6 and the average of 4.08 (±1.27) for known drugs. The KDI_2b_ range was from 0.01 to 0.46, with a theoretical maximum of 1 and with a KDS average of 0.18 (±0.20). When the KDI values of the active ligands were plotted against their IC_50_s, excellent correlations were seen with KDI_2A_ (R^2^—0.859) and KDI_2B_ (R^2^—0.861, see Figures S29 and S30); furthermore, the averages for the active ligands were considerably higher than for the inactive (IC_50_ > 10 µM) derivatives.

In conclusion, activity for the enamine ligands was improved with smaller descriptor values, which fit the modelling observation that only relatively short aliphatic chains are tolerated.

## 4. Discussion

The search for inhibitors of the DNA repair system is a promising area of modern pharmacology that could increase the effectiveness of cancer therapy, especially to drug-resistant tumors. An interesting target for cancer treatment is TDP1, which plays a key role in the removal of DNA damage produced by TOP1 inhibition by clinically important anticancer drugs (irinotecan, Tpc) as well as removal of DNA damage caused by other anticancer drugs.

A literature review of known TDP1 inhibitors showed that the most effective pharmacophoric fragments are of natural products, i.e., a class of phenols (UA [12,13,14,15,24,25]) and terpenes (monoterpenoids [28,29,30,58,59,60]).

As mentioned in the introduction, the addition of a terpene substituent in the TDP1 inhibitor framework leads to a decrease in both effective inhibitory concentration and, in particular, general cytotoxicity. For example, arylidenefuranone compounds inhibit the growth of HEK293 cells in the concentration range of 4.6 to 20 μM [13], while some terpene-furanone derivatives were found to be toxic to these cells from 9 μM [31]. Enamine UA derivatives are generally less cytotoxic than hydrazinothiazole derivatives, e.g., **4** inhibited the growth of MCF-7 cells by half at 1.7 μM [12], and its enamine analog, with a *para*-bromophenyl substituent, at 40 μM [15].

In this study, we synthesized a series of new UA enamino derivatives with terpene fragments, hoping to obtain more effective and less toxic sensitizers to be used with Tpc. The derivatives varied both in the structure of the terpene fragment (linear, monocyclic, bicyclic) and in the linker length. Moreover, these compounds were easier to synthesize in comparison with known UA-based TDP1 inhibitors. Four compounds (**11a**–**d**) exhibited IC_50_ values in the range of 0.23–0.40 μM (Table 1), which was approximately the same as the values for enamines with aryl substituents (0.16–2 μM) [15]. Their analogs with longer substituents **13a**–**d** did not affect the enzyme activity at concentrations of up to 10 μM. Surprisingly, compound **11e** did not inhibit the enzyme, although its substituent did not differ in size from **11d**s substituent, the most effective inhibitor of the entire set. According to molecular modelling, compound **11e** did not form a single hydrogen bond in the allosteric center, in contrast to **11d**, explaining the difference in efficacy.

It was shown by molecular modelling that the UA enamines can occupy both the catalytic and allosteric sites. The effective TDP1 inhibitors **11a–d** have short aliphatic chains and were predicted to adopt a conformation occupying both the catalytic pocket and the allosteric site. The derivatives with long aliphatic chains, with no TDP1 activity at micromolar concentrations (**13a**–**e**), did not fit into both binding domains (Figure 11, C for **11d**). Furthermore, there was a clear correlation of activity of the enamine ligands and smaller descriptor values. This explains why only relatively short aliphatic chains were tolerated. Earlier, we also reported the binding of UA enamines in the allosteric center near the catalytic center, on the basis of molecular dynamics simulations [15].

The intrinsic cytotoxicity of the UA-derived TDP1 inhibitors were checked in two human cell lines, HeLa and HEK293A. The compounds demonstrated low cytotoxicity with CC_50_ values > 60 μM for both cell lines. Low, or insignificant, cytotoxicity of new potential substances for drug combination therapy is important to avoid side effects. By increasing the sensitivity to conventional cytotoxic therapy using a non-toxic agent, it is possible to reduce the toxic load on the patients, making the regime more tolerable. This is very important because many cancer patients are elderly, often with underlying medical conditions, who cannot tolerate an aggressive treatment. We selected two candidates (**11a** and **11c**) for the studies on cell cultures in combination with Tpc on the basis of their TDP1 efficacy and low cytotoxicity. We observed synergistic effect with Tpc for **11a** and **11c**—both compounds enhanced Tpc cytotoxicity on cancerous HeLa cell line. We obtained three TDP1 knockout clones of the HEK293A cell line to confirm that the cellular target of the compounds was TDP1, as previously performed for TDP1 -/- mutants of the HEK293FT line [39]. To our surprise, sensitization was not seen with Tpc in either WT HEK293A cells or TDP1 -/- cells. Adding both Tpc and TDP1 inhibitor (**11a** or **11c**) to HEK293A cells led to the stimulation of growth as compared to only Tpc.

## 5. Conclusions

Cancers are one of the most frequent causes of death in the world. There are several problems arising in the course of chemotherapeutic treatment of oncological diseases, namely, low efficiency of chemotherapy, the resistance of malignant tumors to drugs, many side effects, and a strong toxic load on the body. The cytotoxic effect of chemotherapy is caused by the ability to create DNA damage in cancer cells, and DNA repair is a key mechanism of resistance. The design of new compounds that inhibit DNA repair enzymes is a promising strategy for potentiating the cytotoxicity of DNA damaging agents that are clinically used as therapeutic agents. The enzymes involved in DNA repair, for example, TDP1, are interesting therapeutic targets.

In this work, we chose two compounds (**11a** and **11c**) as favorable candidates for anticancer therapy in combination with Tpc. They were selected out of 10 newly synthesized UA enamino derivatives with terpene fragments on the basis of their TDP1 inhibitory properties and low intrinsic cytotoxicity on HeLa and HEK293A cells. Both compounds enhanced Tpc cytotoxicity on cancerous HeLa cell line but reduced it on non-cancerous HEK293A cells. This “protective” effect from Tpc on non-cancerous cells could be a positive advantage but needs further investigation as well as a search of other cellular protein targets for these UA derivatives.

## Figures and Tables

**Table 1:** NMR ^1^H spectra of **11a**–**e**.

№	a	b	c	d	e
**Structure**	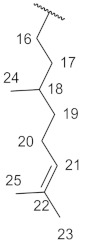	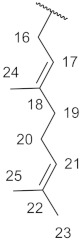	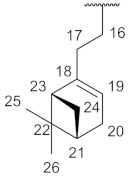	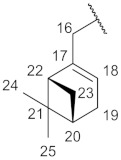	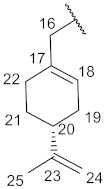
H-16	3.45, m	4.0, m	3.44, m	3.95, m	3.94, m
H-17	1.15–1.52, m	5.28, m	2.18–2.38, m	---	---
H-18	1.73, m	---	---	5.47, ss	5.67, ss
H-19	1.15–1.52, m	2.08, s	5.44, s	2.30, m	1.94–2.20, m
H-20	1.99, m	2.09, s	2.18–2.38, m	2.44, m	1.94–2.20, m
H-21	5.06, m	5.05, m	2.18–2.38, m	---	1.50, m and 1.86, m
H-22	---	---	---	2.0, m	1.94–2.20, m
H-23	1.59, s	1.58, s	2.0, m	1.2 d (8.9) and 2.1, m	---
H-24	0.94, d (6.25)	1.65, s	1.1, d (8.6) and 2.1, m	0.84, s	4.7, d (13.5)
H-25	1.68, s	1.77, s	0.78, s	1.29, s	1.71, s
H-26	---	---	1.25, s	---	---

**Table 2:** NMR ^13^C spectra of **11a–e**.

№	a	b	c	d	e
**Structure**	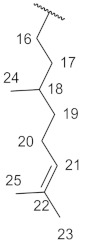	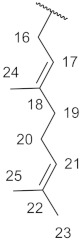	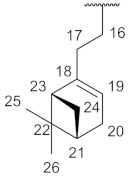	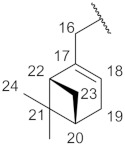	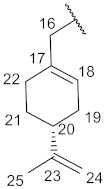
C-16	42.00	41.8	45.2	48.4	49.5
C-17	36.6	117.0	35.7	120.2	148.9
C-18	31.9	142.15	120.4	141.5	125.0
C-19	35.8	39.2	143.0	31.4	30.2
C-20	25.2	26.0	31.2	40.5	40.5
C-21	123.9	123.2	40.5	38.1	26.9
C-22	131.7	132.0	38.0	44.0	27.0
C-23	17.6	25.6	42.0	31.9	131.1
C-24	25.6	16.5	32.0	18.8	108.9
C-25	18.2	17.6	18.4	25.9	18.1
C-26	---	---	26.1	---	---

**Table 3:** NMR ^1^H spectra of **13a**-**e**.

№	a	b	c	d	e
**Structure**	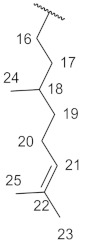	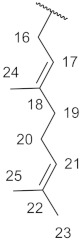	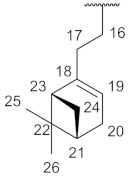	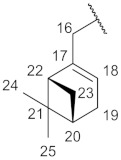	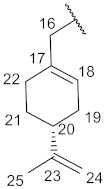
H-16	4.10, m	4.61, m	4.01, m	4.45, m	4.45, m
H-17	1.15−1.52, m	5.31, m	2.10−2.30, m	---	---
H-18	1.68, m	---	---	5.55, m	5.73, s
H-19	1.15−1.52, m	2.09, s	5.27, s	2.25, m	1.86−2.17, m
H-20	1.95, m	2.09, s	2.10−2.30, m	2.38, m	1.86−2.17, m
H-21	5.06, m	5.06, m	2.40, m	---	1.44, m and 1.80, m
H-22	---	---	---	2.0, m	1.86−2.17, m
H-23	1.57, s	1.57, s	2.0, m	1.1 d (8.9)and 2.1, m	---
H-24	0.96, d (6.5)	1.65, s	1.1, d (8.6)and 2.1, m	0.78, s	4.68, m
H-25	1.65, s	1.68, s	0.78, s	1.28, s	1.69, s
H-26	---		1.23, s	---	---

**Table 4:** NMR ^13^C spectra of **13a**–**e**.

№	a	b	c	d	e
**Structure**	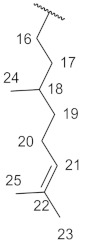	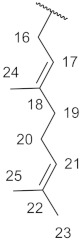	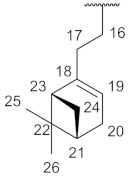	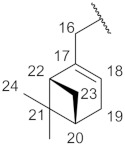	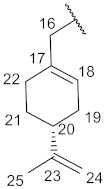
C-16	63.3	61.7	63.0	67.3	68.65
C-17	36.8	117.7	35.7	121.8	149.1
C-18	31.7	142.7	118.8	142.5	126.1
C-19	35.2	39.4	143.7	31.1	30.2
C-20	25.2	26.1	31.2	42.9	40.5
C-21	124.3	123.5	40.5	37.9	26.2
C-22	131.3	132.8	37.8	43.4	27.0
C-23	17.5	25.6	45.4	31.8	132.1
C-24	25.6	16.4	32.0	18.5	108.9
C-25	18.1	17.6	18.2	25.9	18.0
C-26	---	---	26.1	---	---

**Table 5:** The influence of UA derivatives **11a–e** and **13a–e** on TDP1 activity.

Terpene Fragment Structure	Code	IC_50_, µM	Code	IC_50_, µM
	11a–e		13a–e	
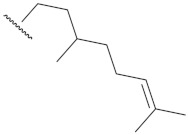	**11a**	0.40 ± 0.09	**13a**	>10
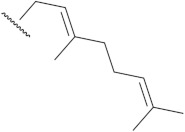	**11b**	0.32 ± 0.13	**13b**	>10
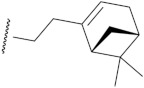	**11c**	0.32 ± 0.10	**13c**	>10
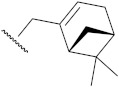	**11d**	0.23 ± 0.10	**13d**	>10
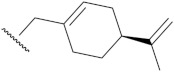	**11e**	>10	**13e**	>10

## Data Availability

The data presented in this study are available on request from the corresponding authors.

## Supplementary Materials

The following are available online at https://www.mdpi.com/article/10.3390/biom11070973/s1, Figures S1–S19: NMR ^13^C spectra of compounds. Figure S20: TDP1 inhibitors’ intrinsic cytotoxicity on HEK293A WT and TDP1 -/- cells (C6, G6, F7), and dose-dependent action of the derivatives **11a, 11b, 11c,** and **11d**. Figure S21: Verification of CRISPR/Cas9-mediated deletions in the TDP1 gene by genome DNA sequencing. Alignment of plasmid clones sequenograms (C6, G6, F7) with the wild-type sequence of the TDP1 gene revealed the presence of deletions that shift the reading frame and potentially disrupt the synthesis of the corresponding protein. Figure S22: Analysis of the HEK293 TDP1 -/- clones. (A) PCR analysis for the presence of CRISPR/Cas9-mediated deletions in TDP1 gene. PCR analysis showed homozygous deletions in the first protein coding exon (third exon in mRNA, NM_001008744.2) of the TDP1 gene in clones C6, G6, and F7 (lanes 3–5). (B) Identification of TDP1 3′-phosphotyrosyl cleavage activity in the HEK293 cell extracts: HEK293FT WT (lane 3); HEK293A WT (lane 4); and clone C6, G6, and F7 cells (lanes 5–7). There was no established cleavage activity in the clone C6, G6, and F7 cell extracts, in contrast to control WT cell extracts and purified TDP1. Figure S23: Topotecan cytotoxicity on HEK293A TDP1 -/- (clones C6, G6, and F7) cells, and dose-dependent action of topotecan in combination with **11a** or **11c** compounds by colorimetric test. Figure S24: Topotecan cytotoxicity on HeLa cells, and dose-dependent action of topotecan in combination with **11a** (left) or **11c** (right) compounds by colorimetric test and CompuSyn version 1.0 software. Table S1: The binding affinities as predicted by the scoring functions used to the catalytic binding site. Table S2: The binding affinities as predicted by the scoring functions used to the allosteric binding site. Table S3: The molecular descriptors and their corresponding Known Drug Indexes 2a and 2b (KDI_2a/2b_). The R^2^ numbers derived do not contain the IC_50_ > 10 µM values. Table S4: Definition of lead-like, drug-like, and Known Drug Space (KDS) in terms of molecular descriptors. The values given are the maxima for each descriptor for the volumes of chemical space used. Figure S25: The correlation of the IC_50_ values of the active ligands with RB. Figure S26: The correlation of the IC_50_ values of the active ligands with MW. **11c** was an outlier and was not included in the R^2^. Figure S27: The correlation of the IC_50_ values of the active ligands with Log P. Figure S28: The correlation of the IC_50_ values of the active ligands with PSA. Figure S28: The correlation of the IC_50_ values of the active ligands with KDI_2A_. Figure S30: The correlation of the IC_50_ values of the active ligands with KDI_2B_.

## Abbreviations

TDP1	tyrosyl-DNA phosphodiesterase 1:
TG7	4-[(2-phenylimidazo[1,2-a]pyridin-3-yl)amino]benzene-1,2-dicarboxylic acid;
TOP1	DNA-topoisomerase 1;
Tpc	topotecan;
UA	usnic acid;
GABA	γ-aminobutyricacid;
EDC	1-(3-dimethylamino-propyl)-3-ethylcarbodiimide hydrochloride;
DMAP	dimethylaminopyridine.

## References

[1] Ingolfsdottir K. (2002). Usnic acid. Phytochemistry.

[2] Luzina O.A., Salakhutdinov N.F. (2018). Usnic acid and its derivatives for pharmaceutical use: A patent review (2000–2017). Expert Opin. Ther. Pat..

[3] Neff G.W., Reddy K.R., Durazo F.A., Meyer D., Marrero R., Kaplowitz N. (2004). Severe hepatotoxicity associated with the use of weight loss diet supplements containing ma huang or usnic acid. J. Hepatol..

[4] Sviridov V.N., Strigina L.I. (1976). (+)-Usnic acid in Far Eastern lichens. Chem. Nat. Compd..

[5] Galanty A., Pasko P., Podolak I. (2019). Enantioselective activity of usnic acid: A comprehensive review and future perspectives. Phytochem. Rev..

[6] Bekker O.B., Sokolov D.N., Luzina O.A., Komarova N.I., Gatilov Y.V., Andreevskaya S.N., Smirnova T.G., Maslov D.A., Chernousova L.N., Salakhutdinov N.F. (2015). Synthesis and activity of (+)-usnic acid and (-)-usnic acid derivatives containing 1,3-thiazole cycle against Mycobacterium tuberculosis. Med. Chem. Res..

[7] Shtro A.A., Zarubaev V.V., Luzina O.A., Sokolov D.N., Salakhutdinov N.F. (2015). Derivatives of usnic acid inhibit broad range of influenza viruses and protect mice from lethal influenza infection. Antivir. Chem. Chemother..

[8] Shtro A.A., Zarubaev V.V., Luzina O.A., Sokolov D.N., Kiselev O.I., Salakhutdinov N.F. (2014). Novel derivatives of usnic acid effectively inhibiting reproduction of influenza A virus. Bioorg. Med. Chem..

[9] Krukov V.Y., Tomilova O.G., Luzina O.A., Yaroslavtseva O.N., Akhanaev Y.B., Tyurin M.V., Duisembekov B.A., Salakhutdinov N.F., Glupov V.V. (2018). Effects of fluorine-containing usnic acid and fungus Beauveria bassiana on the survival and immune-physiological reactions of Colorado potato beetle larvae. Pest Manag. Sci..

[10] Luzina O.A., Salakhutdinov N.F. (2016). Biological activity of usnic acid and its derivatives: Part 1. Activity against unicellular organisms. Russ. J. Bioorg. Chem..

[11] Luzina O.A., Salakhutdinov N.F. (2016). Biological activity of usnic acid and its derivatives: Part 2. effects on higher organisms. Molecular and physicochemical aspects. Russ. J. Bioorg. Chem..

[12] Zakharenko A.L., Luzina O.A., Sokolov D.N., Kaledin V.I., Nikolin V.P., Popova N.A., Patel J., Zakharova O.D., Chepanova A.A., Zafar A. (2019). Novel tyrosyl-DNA phosphodiesterase 1 inhibitors enhance the therapeutic impact of topotecan on in vivo tumor models. Eur. J. Med. Chem..

[13] Zakharova O., Luzina O., Zakharenko A., Sokolov D., Filimonov A., Dyrkheeva N., Chepanova A., Ilina E., Ilyina A., Klabenkova K. (2018). Synthesis and evaluation of aryliden- and hetarylidenfuranone derivatives of usnic acid as highly potent Tdp1 inhibitors. Bioorg. Med. Chem..

[14] Zakharenko A.L., Luzina O.A., Sokolov D.N., Zakharova O.D., Rakhmanova M.E., Chepanova A.A., Dyrkheeva N.S., Lavrik O.I., Salakhutdinov N.F. (2017). Usnic Acid Derivatives Are Effective Inhibitors of Tyrosyl-DNA Phosphodiesterase 1. Russ. J. Bioorg. Chem..

[15] Zakharenko A., Luzina O., Koval O., Nilov D., Gushchina I., Dyrkheeva N., Švedas V., Salakhutdinov N., Lavrik O. (2016). Tyrosyl-DNA Phosphodiesterase 1 Inhibitors: Usnic Acid Enamines Enhance the Cytotoxic Effect of Camptothecin. J. Nat. Prod..

[16] Comeaux E.Q., van Waardenburg R.C. (2014). Tyrosyl-DNA phosphodiesterase I resolves both naturally and chemically induced DNA adducts and its potential as a therapeutic target. Drug Metab. Rev..

[17] Kawale A.S., Povirk L.F. (2018). Tyrosyl-DNA phosphodiesterases: Rescuing the genome from the risks of relaxation. Nucleic Acids Res..

[18] Pommier Y., Leo E., Zhang H., Marchand C. (2010). DNA topoisomerases and their poisoning by anticancer and antibacterial drugs. Chem Biol..

[19] Pommier Y. (2004). Camptothecins and topoisomerase I: A foot in the door. Targeting the genome beyond topoisomerase I with camptothecins and novel anticancer drugs: Importance of DNA replication, repair and cell cycle checkpoints. Curr. Med. Chem. Anticancer Agents.

[20] Beretta G.L., Cossa G., Gatti L., Zunino F., Perego P. (2010). Tyrosyl-DNA phosphodiesterase 1 targeting for modulation of camptothecin-based treatment. Curr. Med. Chem..

[21] Alagoz M., Gilbert D.C., El-Khamisy S., Chalmers A.J. (2012). DNA repair and resistance to topoisomerase I inhibitors: Mechanisms, biomarkers and therapeutic targets. Curr. Med. Chem..

[22] Laev S.S., Salakhutdinov N.F., Lavrik O.I. (2016). Tyrosyl-DNA phosphodiesterase inhibitors: Progress and potential. Bioorg. Med. Chem..

[23] Thomas A., Pommier Y. (2019). Targeting Topoisomerase I in the Era of Precision Medicine. Clin. Cancer Res..

[24] Filimonov A.S., Chepanova A.A., Luzina O.A., Zakharenko A.L., Zakharova O.D., Ilina E.S., Dyrkheeva N.S., Kuprushkin M.S., Kolotaev A.V., Khachatryan D.S. (2019). New Hydrazinothiazole Derivatives of Usnic Acid as Potent Tdp1 Inhibitors. Molecules.

[25] Koldysheva E.V., Men’shchikova A.P., Lushnikova E.L., Popova N.A., Kaledin V.I., Nikolin V.P., Zakharenko A.L., Luzina O.A., Salakhutdinov N.F., Lavrik O.I. (2019). Antimetastatic Activity of Combined Topotecan and Tyrosyl-DNA Phosphodiesterase-1 Inhibitor on Modeled Lewis Lung Carcinoma. Bull. Exp. Biol. Med..

[26] Dyrkheeva N.S., Zakharenko A.L., Novoselova E.S., Chepanova A.A., Popova N.A., Nikolin V.P., Luzina O.A., Salakhutdinov N.F., Ryabchikova E.I., Lavrik O.I. (2021). Antitumor Activity of the Combination of Topotecan and Tyrosyl-DNA-Phosphodiesterase 1 Inhibitor on Model Krebs-2 Mouse Ascite Carcinoma. Mol. Biol..

[27] Salakhutdinov N.F., Volcho K.P., Yarovaya O.I. (2017). Monoterpenes as a renewable source of biologically active compounds. Pure Appl. Chem..

[28] Khomenko T., Zakharenko A., Odarchenko T., Arabshahi H.J., Sannikova V., Zakharova O., Korchagina D., Reynisson J., Volcho K., Salakhutdinov N. (2016). New inhibitors of tyrosyl-DNA phosphodiesterase I (Tdp 1) combining 7-hydroxycoumarin and monoterpenoid moieties. Bioorg. Med. Chem..

[29] Munkuev A.A., Mozhaitsev E.S., Chepanova A.A., Suslov E.V., Korchagina D.V., Zakharova O.D., Ilina E.S., Dyrkheeva N.S., Zakharenko A.L., Reynisson J. (2021). Novel Tdp1 inhibitors based on adamantane connected with monoterpene moieties via heterocyclic fragments. Molecules.

[30] Khomenko T.M., Zakharenko A.L., Chepanova A.A., Ilina E.S., Zakharova O.D., Kaledin V.I., Nikolin V.P., Popova N.A., Korchagina D.V., Reynisson J. (2019). Promising New Inhibitors of Tyrosyl-DNA Phosphodiesterase I (Tdp 1) Combining 4-Arylcoumarin and Monoterpenoid Moieties as Components of Complex Antitumor Therapy. Int. J. Mol. Sci..

[31] Dyrkheeva N., Luzina O., Filimonov A., Zakharova O., Ilina E., Zakharenko A., Kuprushkin M., Nilov D., Gushchina I., Švedas V. (2019). Inhibitory Effect of New Semisynthetic Usnic Acid Derivatives on Human Tyrosyl-DNA Phosphodiesterase 1. Planta Med..

[32] Luzina O., Filimonov A., Zakharenko A., Chepanova A., Zakharova O., Ilina E., Dyrkheeva N., Likhatskaya G., Salakhutdinov N., Lavrik O. (2020). Usnic Acid Conjugates with Monoterpenoids as Potent Tyrosyl-DNA Phosphodiesterase 1 Inhibitors. J. Nat. Prod..

[33] Polovinka M.P., Salakhutdinov N.F., Panchenko M.Y. (2008). Method for obtaining usnic acid.

[34] Luzina O.A., Polovinka M.P., Salakhutdinov N.F., Tolstikov G.A. (2007). Chemical modification of usnic acid 2. Reactions of (+)-usnic acid with amino acids. Russ. Chem. Bull..

[35] Suslov E.V., Mozhaytsev E.S., Korchagina D.V., Bormotov N.I., Yarovaya O.I., Volcho K.P., Salakhutdinov N.F. (2020). New chemical agents based on adamantane–monoterpene conjugates against orthopoxvirus infections. RSC Med. Chem..

[36] Zakharenko A., Khomenko T., Zhukova S., Koval O., Zakharova O., Anarbaev R., Lebedeva N., Korchagina D., Komarova N., Vasiliev V. (2015). Synthesis and biological evaluation of novel tyrosyl-DNA phosphodiesterase 1 inhibitors with a benzopentathiepine moiety. Bioorg. Med. Chem..

[37] Dyrkheeva N., Anarbaev R., Lebedeva N., Kuprushkin M., Kuznetsova A., Kuznetsov N., Rechkunova N., Lavrik O. (2020). Human Tyrosyl-DNA Phosphodiesterase 1 Possesses Transphosphooligonucleotidation Activity with Primary Alcohols. Front. Cell Dev. Biol..

[38] Interthal H., Chen H.J., Champoux J.J. (2005). Human Tdp1 cleaves a broad spectrum of substrates, including phosphoamide linkages. J. Biol. Chem..

[39] Il’ina I.V., Dyrkheeva N.S., Zakharenko A.L., Sidorenko A.Y., Li-Zhulanov N.S., Korchagina D.V., Chand R., Ayine-Tora D.M., Chepanova A.A., Zakharova O.D. (2020). Design, Synthesis, and Biological Investigation of Novel Classes of 3-Carene-Derived Potent Inhibitors of TDP1. Molecules.

[40] Zhao X.Z., Kiselev E., Lountos G.T., Wang W., Tropea J.E., Needle D., Hilimire T.A., Schneekloth J.S., Waugh D.S., Pommier Y. (2021). Small Molecule Microarray Identifies Inhibitors of Tyrosyl-DNA Phosphodiesterase 1 that Simultaneously Access the Catalytic Pocket and Two Substrate Binding Sites. Chem. Sci..

[41] Berman H.M., Westbrook J., Feng Z., Gilliland G., Bhat T.N., Weissig H., Shindyalov I.N., Bourne P.E. (2000). The Protein Data Bank. Nuc. Acids Res..

[42] Berman H., Henrick K., Nakamura H. (2003). Announcing the Worldwide Protein Data Bank. Nat. Struct. Biol..

[43] Scigress Ultra V.F. (2008–2016). J 2.6. (EU 3.1.7).

[44] Allinger N.L. (1977). Conformational Analysis. 130. MM2. A Hydrocarbon Force Field Utilizing V1 and V2 Torsional Terms. J. Am. Chem. Soc..

[45] Gotō H., Ōsawa E. (1993). An Efficient Algorithm for Searching Low-Energy Conformers of Cyclic and Acyclic Molecules. J. Chem. Soc., Perkin Trans..

[46] Jones G., Willet P., Glen R.C., Leach A.R., Taylor R. (1997). Development and Validation of a Genetic Algorithm for Flexible Docking. J. Mol. Biol..

[47] Eldridge M.D., Murray C., Auton T.R., Paolini G.V., Mee P.M. (1997). Empirical Scoring Functions: I. the Development of a Fast Empirical Scoring Function to Estimate the Binding Affinity of Ligands in Receptor Complexes. J. Comp. Aid. Mol. Design.

[48] Verdonk M.L., Cole J.C., Hartshorn M.J., Murray C.W., Taylor R.D. (2003). Improved Protein-Ligand Docking using GOLD. Proteins.

[49] Korb O., Stützle T., Exner T.E. (2009). Empirical Scoring Functions for Advanced Protein−Ligand Docking with PLANTS. J. Chem. Inf. Model..

[50] Mooij W.T.M., Verdonk M.L. (2005). General and Targeted Statistical Potentials for Protein–ligand Interactions. Proteins.

[51] (2021). QikProp, Version 6.2.

[52] Ioakimidis L., Thoukydidis L., Naeem S., Mirza A., Reynisson J. (2008). Benchmarking the Reliability of QikProp. Correlation between Experimental and Predicted Values. QSAR Comb. Sci..

[53] Eurtivong C., Reynisson J. (2018). The Development of a Weighted Index to Optimise Compound Libraries for High Throughput Screening. Mol. Inf..

[54] Sheehan J.C., Bolhofer V.A. (1950). An Improved Procedure for the Condensation of Potassium Phthalimide with Organic Halides. J. Am. Chem. Soc..

[55] Zhu F., Logan G., Reynisson J. (2012). Wine Compounds as a Source for HTS Screening Collections. A Feasibility Study. Mol. Inf..

[56] Salomatina O.V., Popadyuk I.I., Zakharenko A.L., Zakharova O.D., Chepanova A.A., Dyrkheeva N.S., Komarova N.I., Reynisson J., Anarbaev R.O., Salakhutdinov N.F. (2021). Deoxycholic Acid as a Molecular Scaffold for Tyrosyl-DNA Phosphodiesterase 1 Inhibition: A Synthesis, Structure–activity Relationship and Molecular Modeling Study. Steroids.

[57] Gladkova E.D., Chepanova A.A., Ilina E.S., Zakharenko A.L., Reynisson J., Luzina O.A., Volcho K.P., Lavrik O.I., Salakhutdinov N.F. (2021). Discovery of Novel Sultone Fused Berberine Derivatives as Promising TDP1 Inhibitors. Molecules.

[58] Chepanova A.A., Mozhaitsev E.S., Munkuev A.A., Suslov E.V., Korchagina D.V., Zakharova O.D., Zakharenko A.L., Patel J., Ayine-Tora D.M., Reynisson J. (2019). The Development of Tyrosyl-DNA Phosphodiesterase 1 Inhibitors. Combination of Monoterpene and Adamantine Moieties via Amide or Thioamide Bridges. Appl. Sci..

[59] Mozhaitsev E., Suslov E., Demidova Y., Korchagina D., Volcho K., Zakharenko A., Vasi’eva I., Kupryushkin M., Chepanova A., Ayine-Tora D.M. (2019). The Development of Tyrosyl-DNA Phosphodyesterase 1 (TDP1) Inhibitors Based on the Amines Combining Aromatic/Heteroaromatic and Monoterpenoid Moieties. Lett. Drug Des. Discov..

[60] Mozhaitsev E.S., Zakharenko A.L., Suslov E.V., Korchagina D.V., Zakharova O.D., Vasil’eva I.A., Chepanova A.A., Black E., Patel J., Chand R. (2019). Novel Inhibitors of DNA Repair Enzyme TDP1 Combining Monoterpenoid and Adamantane Fragments. Anticancer Agents Med. Chem..

