# Supplementary material for: New Hybrid Compounds Combining Fragments of Usnic Acid and Monoterpenoids for Effective Tyrosyl-DNA Phosphodiesterase 1 Inhibition
Source: Biomolecules. 2021 Jul 1;11(7):973. doi: 10.3390/biom11070973 (PMC8301776; doi:10.3390/biom11070973)
Supplement: Supplementary file 1 [file biomolecules-11-00973-s001.zip › biomolecules-1258269-supplementary.pdf]

Supplementary Materials

New Hybrid Compounds Combining Fragments of Usnic Acid and Monoterpenoids for Effective Tyrosyl-DNA Phosphodiesterase 1 Inhibition

Nadezhda S. Dyrkheeva [1], Aleksandr S. Filimonov [2], Olga A. Luzina [2], Alexandra L. Zakharenko [1], Ekaterina S. Ilina [1], Anastasia A. Malakhova [1,4], Sergey P. Medvedev [1,4], Jóhannes Reynisson [5], Konstantin P. Volcho [2,*], Suren M. Zakian [1,4], Nariman F. Salakhutdinov [2], Olga I. Lavrik [1,3,*]

[1] Institute of Chemical Biology and Fundamental Medicine, Siberian Branch of the Russian Academy of Sciences, 630090 Novosibirsk, Russia; dyrkheeva.n.s@gmail.com (N.S.D.); a.zakharenko73@gmail.com (A.L.Z.); katya.plekhanova@gmail.com (E.S.I.); amal@bionet.nsc.ru (A.A.M.); medvedev@bionet.nsc.ru (S.P.M.); zakian@bionet.nsc.ru (S.M.Z.)
[2] N.N. Vorozhtsov Novosibirsk Institute of Organic Chemistry, Siberian Branch of the Russian Academy of Sciences, 630090 Novosibirsk, Russia; alfil@nioch.nsc.ru (A.S.F.); luzina@nioch.nsc.ru (O.A.L.); anvar@nioch.nsc.ru (N.F.S.)
[3] Federal Research Centre Institute of Cytology and Genetics, Siberian Branch of the Russian Academy of Sciences, 630090 Novosibirsk, Russia
[4] School of Pharmacy and Bioengineering, Keele University, Hornbeam Building, Staffordshire ST5 5BG, UK; j.reynisson@keele.ac.uk
[5] Department of Natural Sciences, Novosibirsk State University, 630090 Novosibirsk, Russia
* Correspondence: volcho@nioch.nsc.ru (K.P.V.); lavrik@niboch.nsc.ru (O.I.L.)

Table of contents:

Figures S1-S19. ^{1}H and ^{13}C NMR spectra of compounds **11a-e** and **13a-e.**

Figure S20. TDP1 inhibitors' intrinsic cytotoxicity on HEK293A WT and TDP1 -/- cells (C6, G6, F7), dose-dependent action of the derivatives **11a, 11b, 11c, 11d**.

Figure S21. Verification of CRISPR/Cas9-mediated deletions in the TDP1 gene by genome DNA sequencing.

Figure S22. Analysis of the HEK293 TDP1 -/- clones.

Figure S23. Topotecan cytotoxicity on HEK293A TDP1 -/- (clones C6, G6, F7) cells, dose-dependent action of topotecan in combination with 11a or 11c compounds by colorimetric test.

Figure S24. Topotecan cytotoxicity on HeLa cells, dose-dependent action of topotecan in combination with **11a** (left) or **11c** (right) compounds by colorimetric test and CompuSyn version 1.0 software.

Table S1. The binding affinities as predicted by the scoring functions used to the catalytic binding site.

Table S2. The binding affinities as predicted by the scoring functions used to the allosteric binding site.

Table S3. The molecular descriptors and their corresponding Known Drug Indexes 2a and 2b ($KDI_{2a/2b}$). The R^2 numbers derived do not contain the IC_{50} > 10 μM values.

Table S4. Definition of lead-like, drug-like and Known Drug Space (KDS) in terms of molecular descriptors. The values given are the maxima for each descriptor for the volumes of chemical space used.

Figure S25. The correlation of the IC_{50} values of the active ligands with RB.

Figure S26. The correlation of the IC_{50} values of the active ligands with MW. 11c is an outlier and not included in the R^2.

Figure S27. The correlation of the IC_{50} values of the active ligands with Log P.

Figure S28. The correlation of the IC_{50} values of the active ligands with PSA.

Figure S29. The correlation of the IC_{50} values of the active ligands with KDI_{2A}.

Figure S30. The correlation of the IC_{50} values of the active ligands with KDI_{2B}.

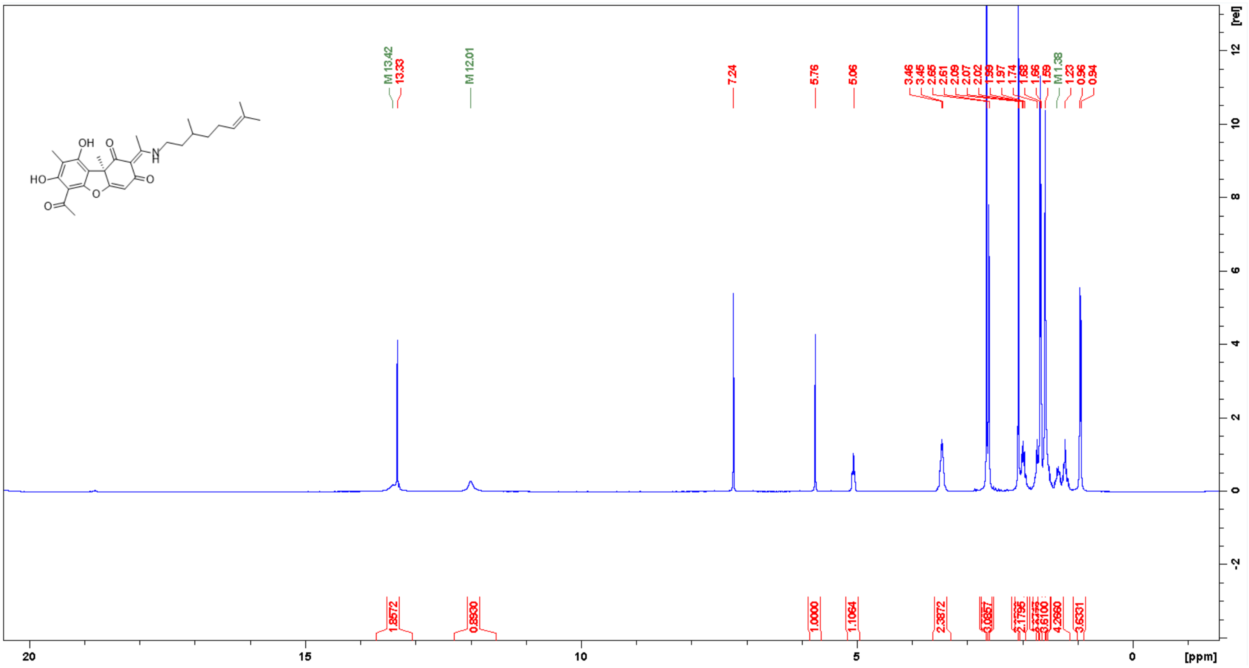

Figure S1. NMR ^{1}H spectra of **11a.**

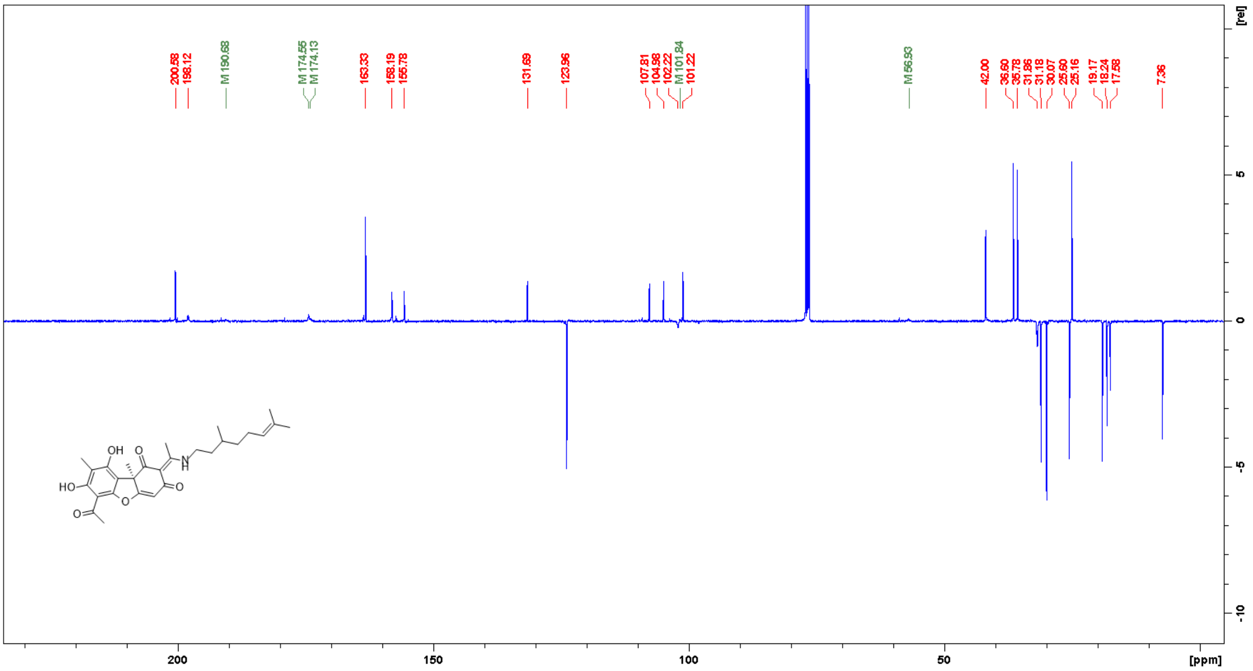

Figure S2. NMR ^{13}C (J-MOD) spectra of **11a.**

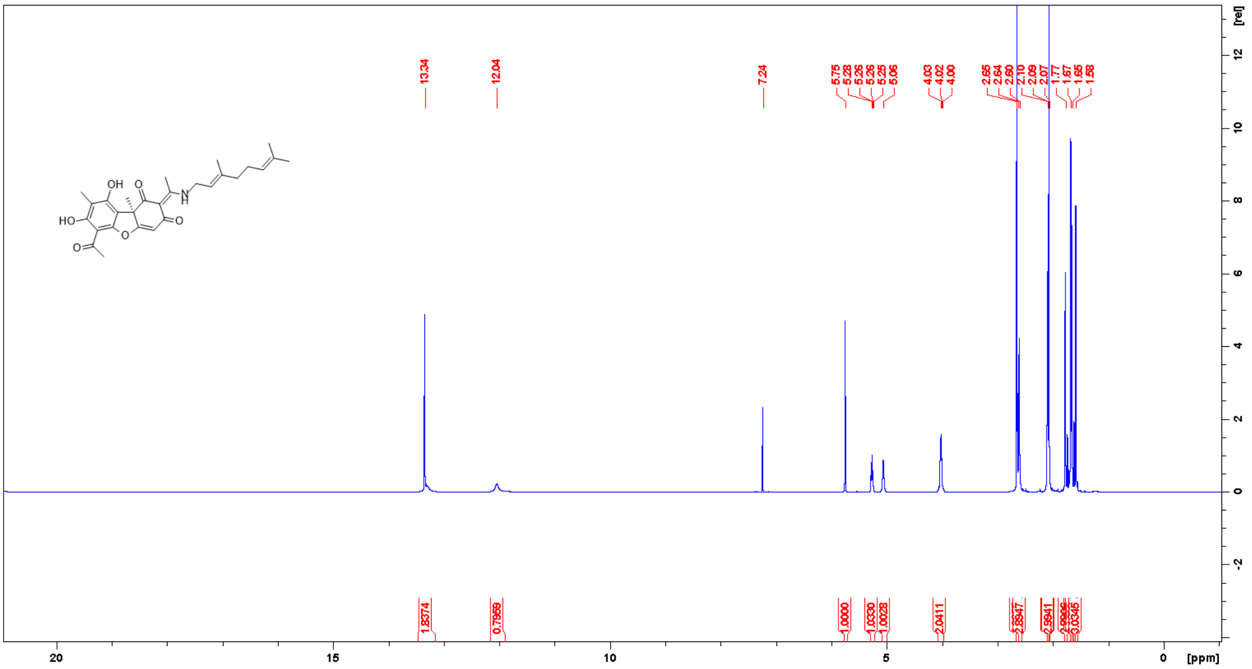

Figure S3. NMR ^{1}H spectra of **11b.**

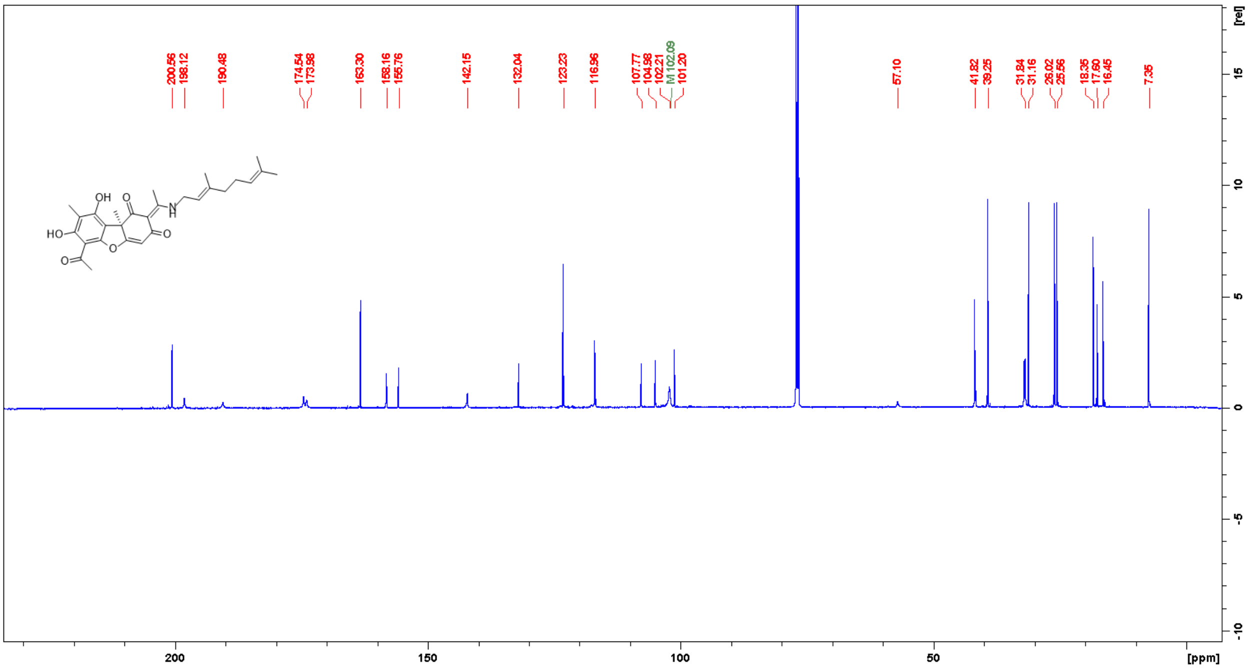

Figure S4. NMR ^{13}C spectra of **11b.**

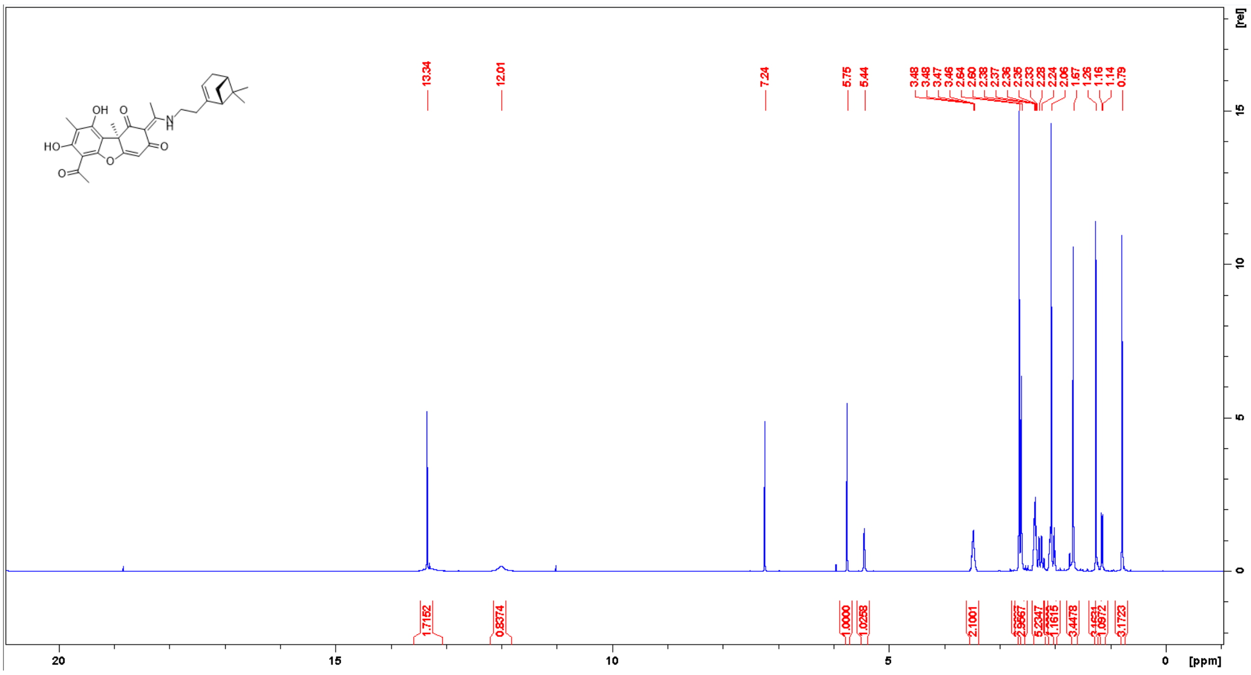

Figure S5. NMR ^{1}H spectra of **11c.**

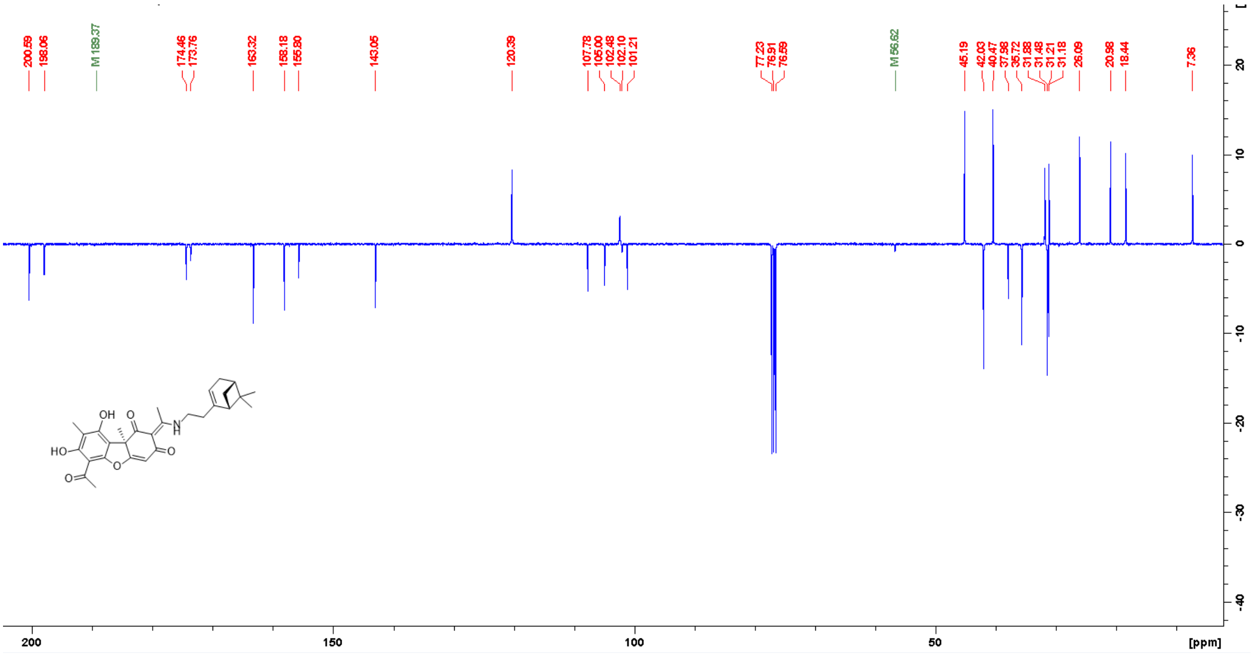

Figure S6. NMR ^{13}C (J-MOD) spectra of **11c.**

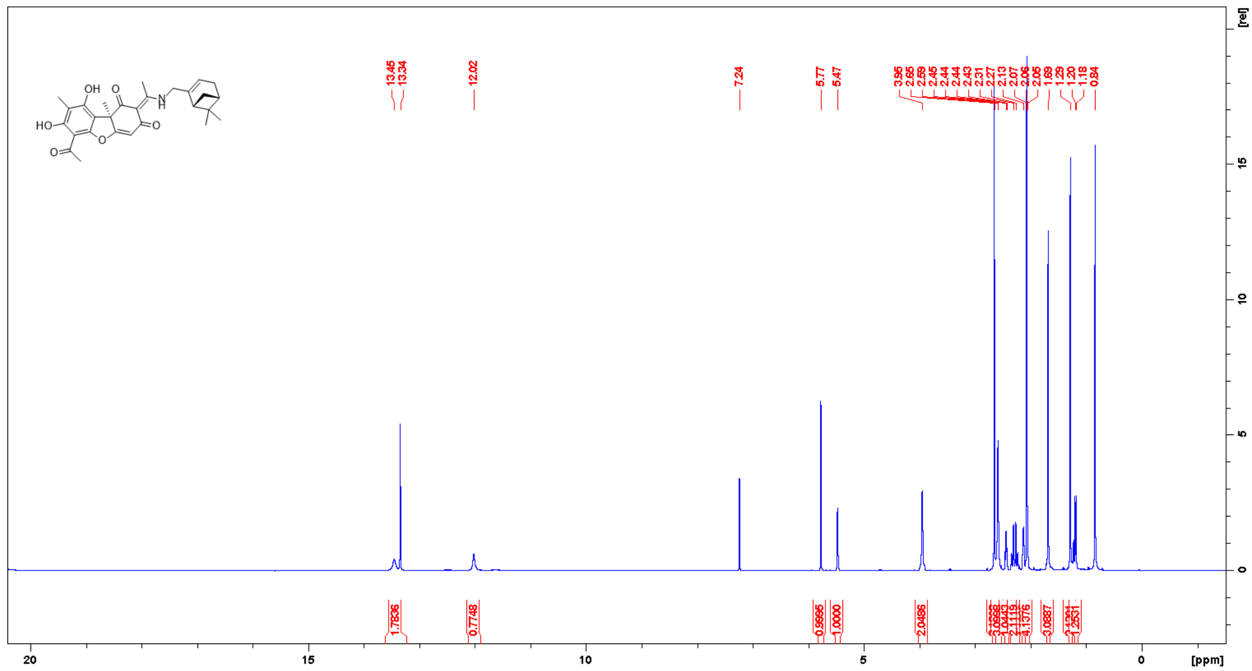

Figure S7. NMR ^{1}H spectra of **11d.**

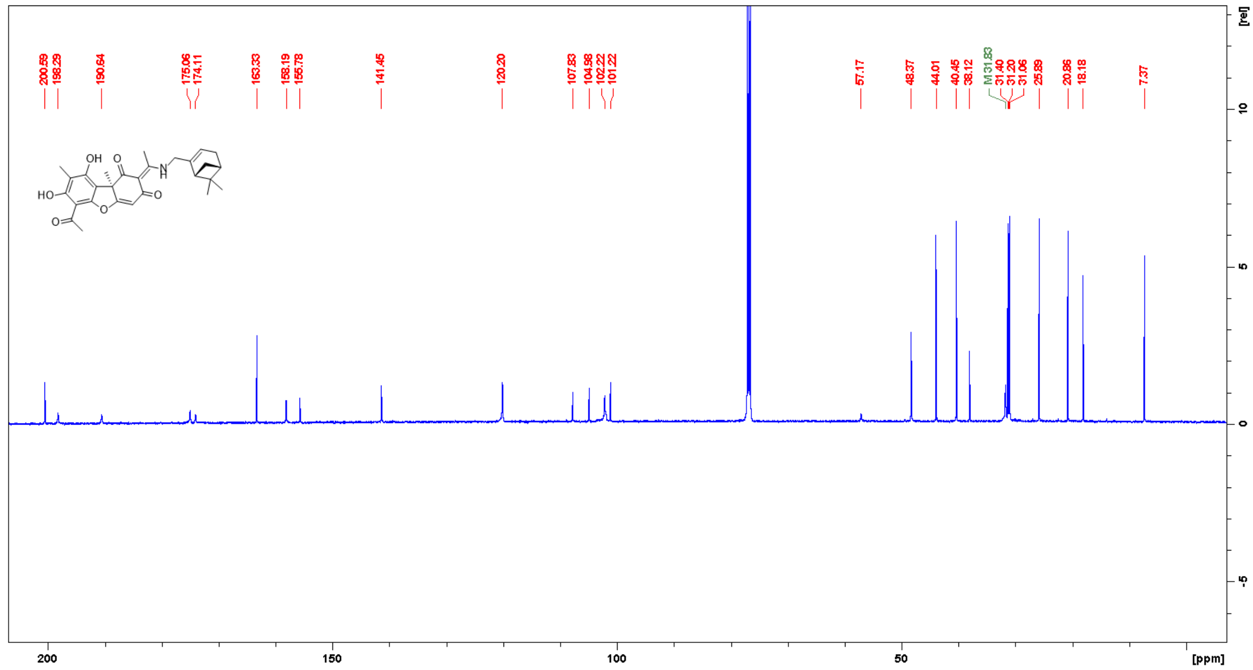

Figure S8. NMR ^{13}C (J-MOD) spectra of **11d.**

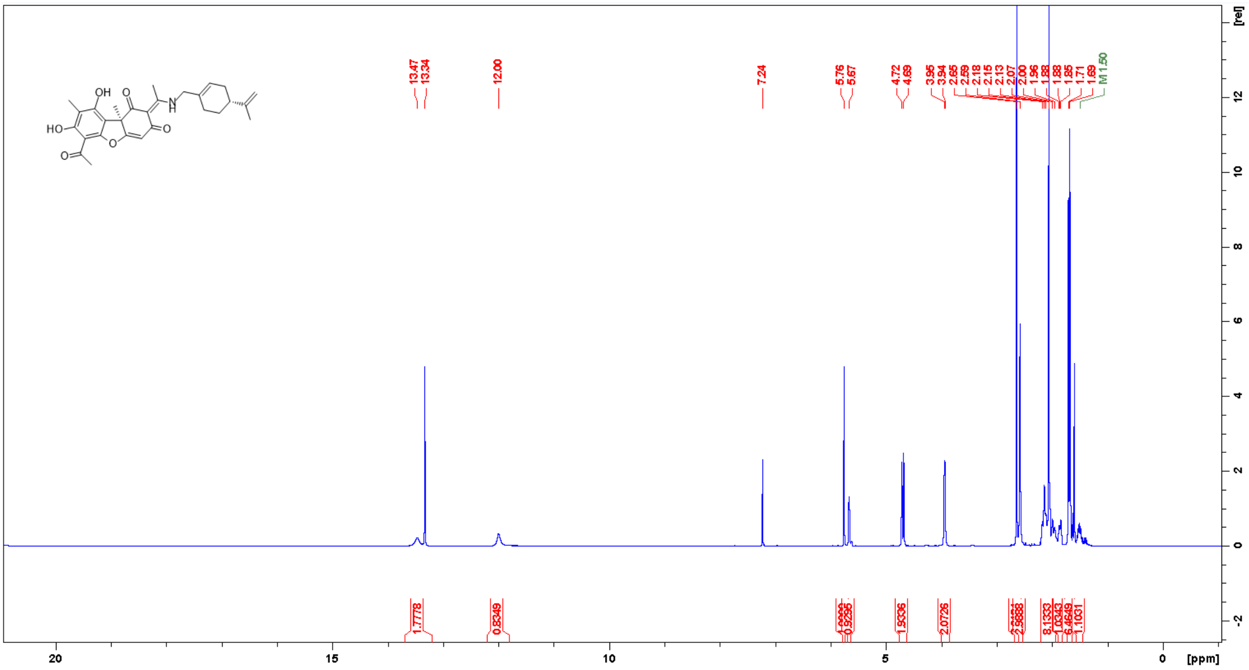

Figure S9. NMR ^{1}H spectra of **11e.**

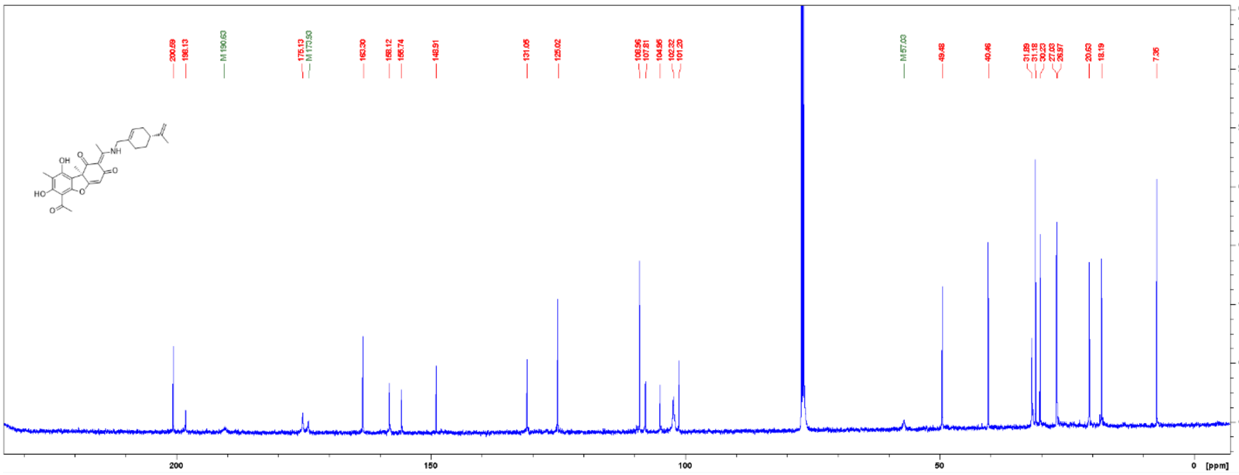

Figure S10. NMR ^{13}C spectra of **11e.**

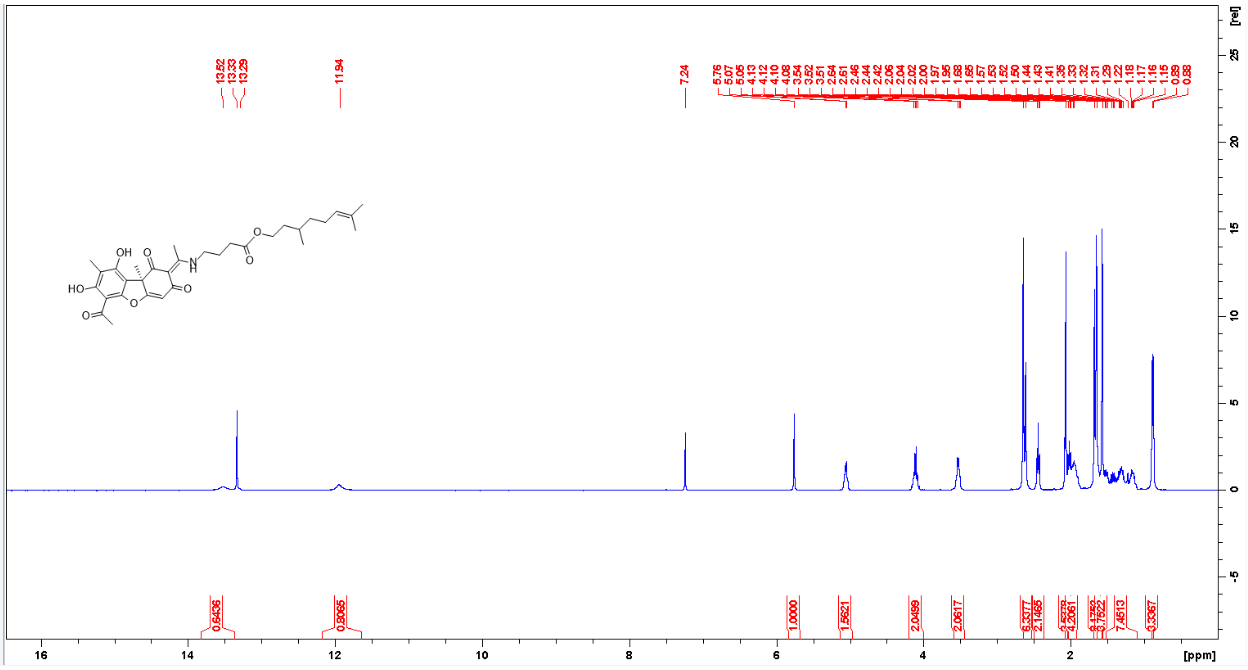

Figure S11. NMR ^{1}H spectra of **13a.**

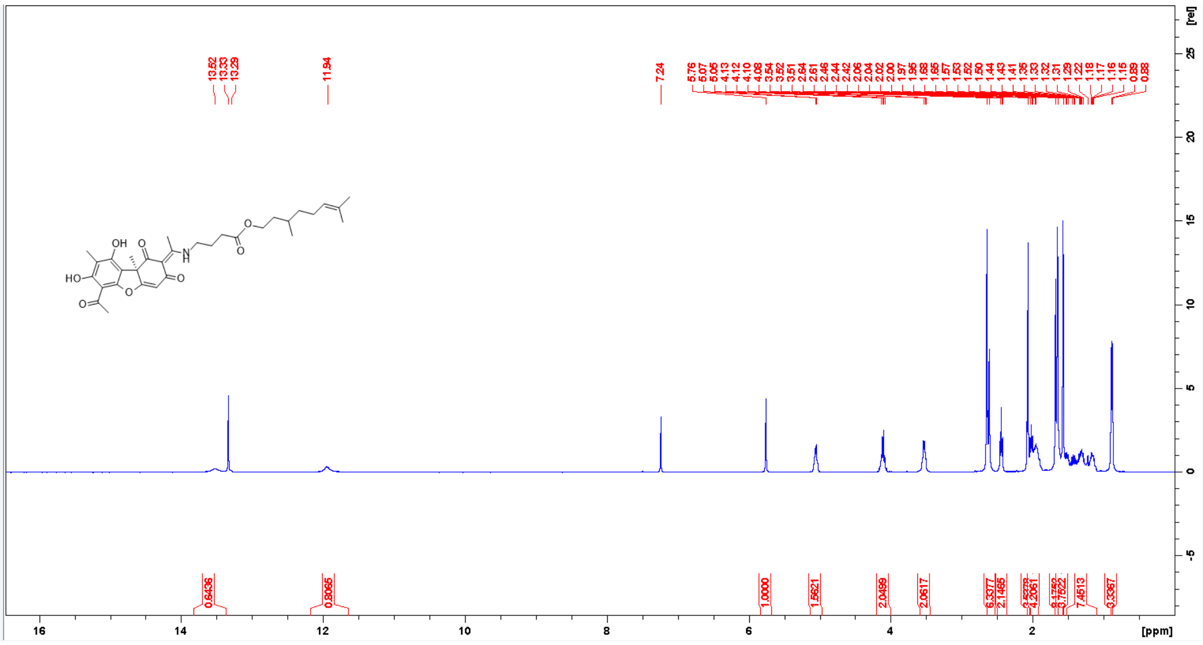

Figure S12. NMR ^{13}C spectra of **13a.**

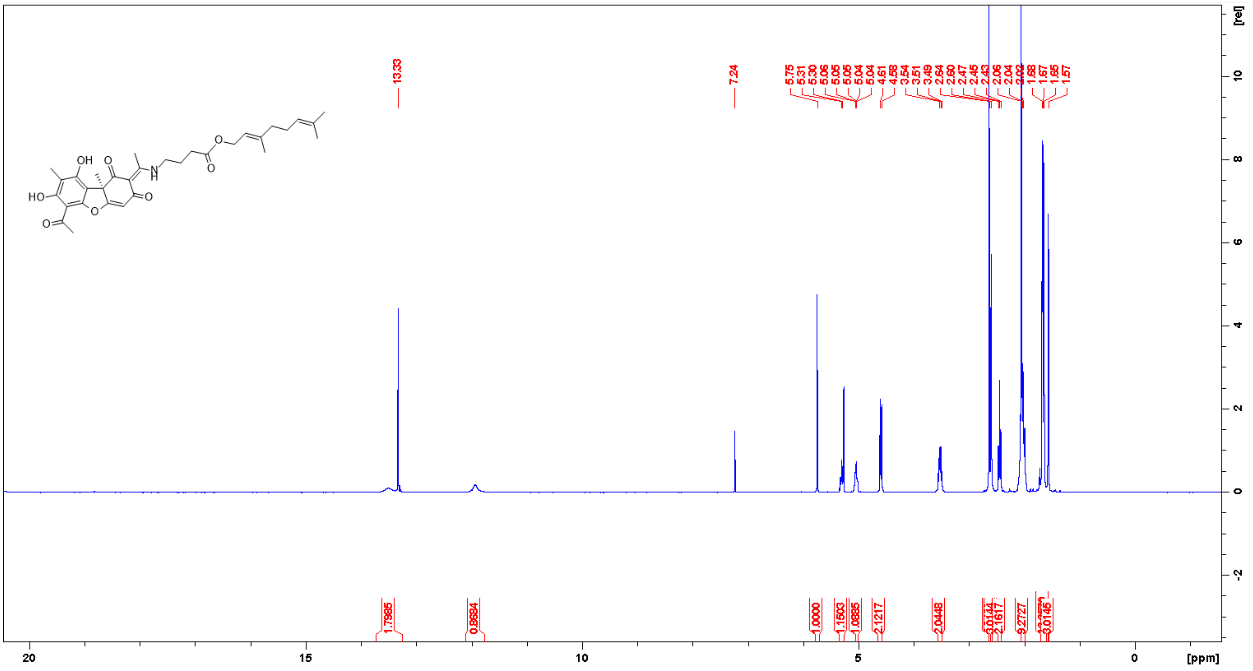

Figure S13. NMR ^{1}H spectra of **13b.**

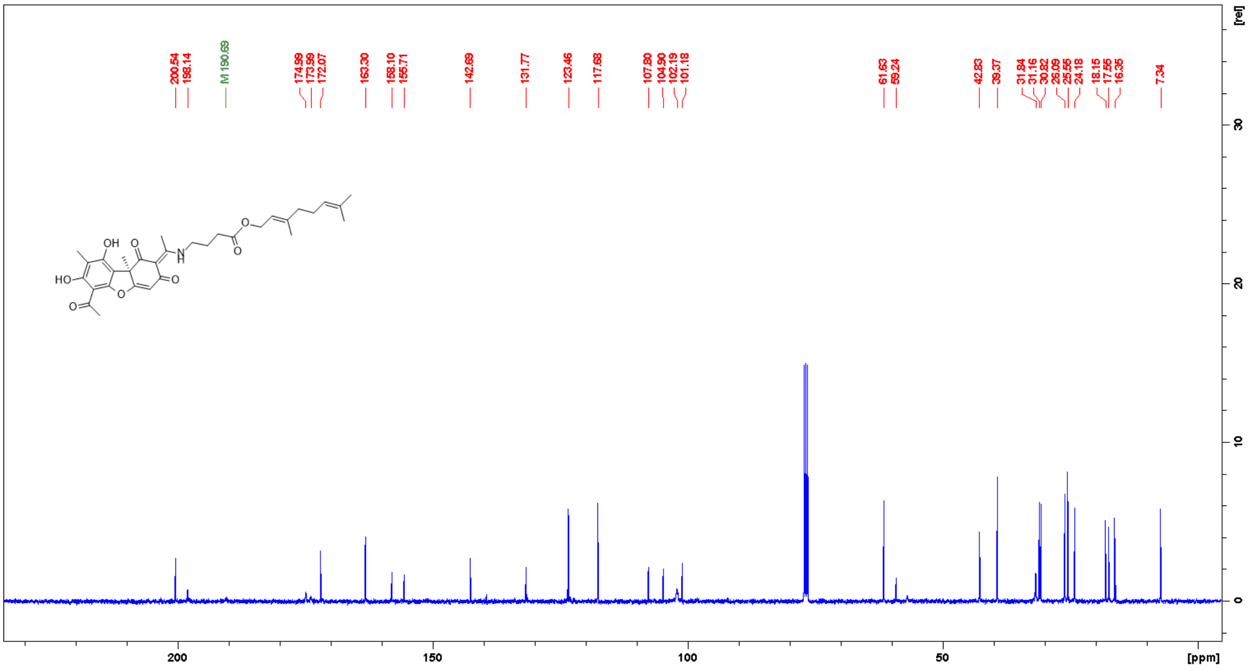

Figure S14. NMR ^{13}C spectra of **13b.**

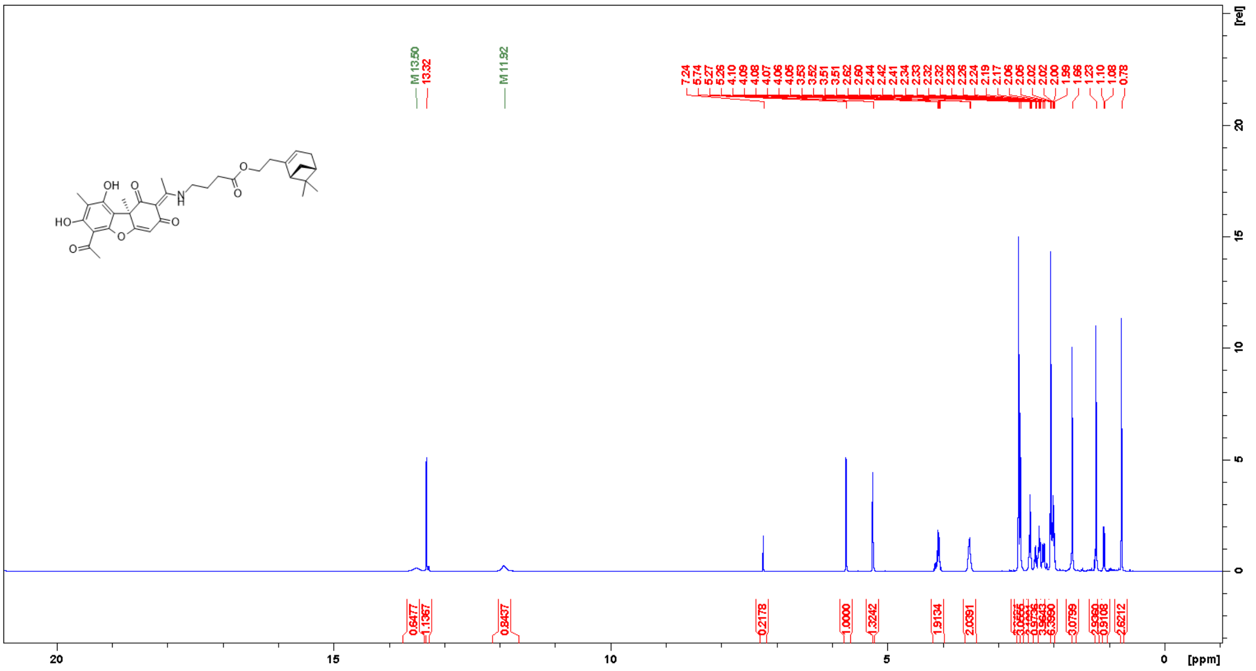

Figure S15. NMR ^{1}H spectra of **13c.**

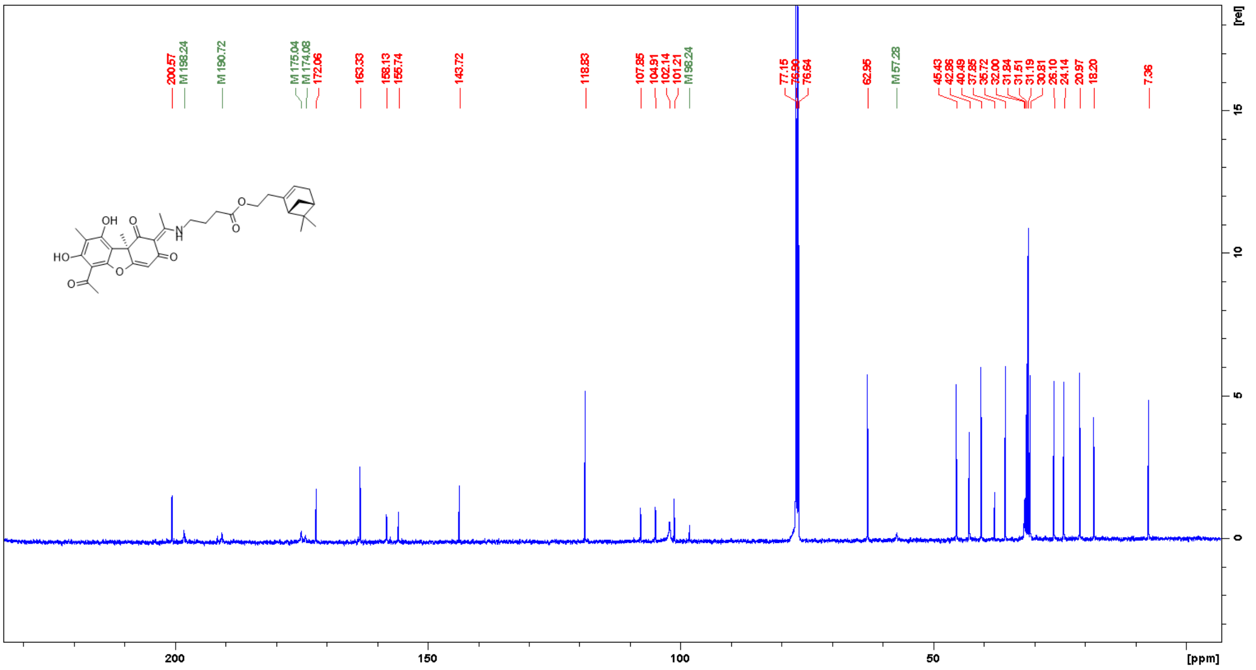

Figure S16. NMR ^{13}C spectra of **13c.**

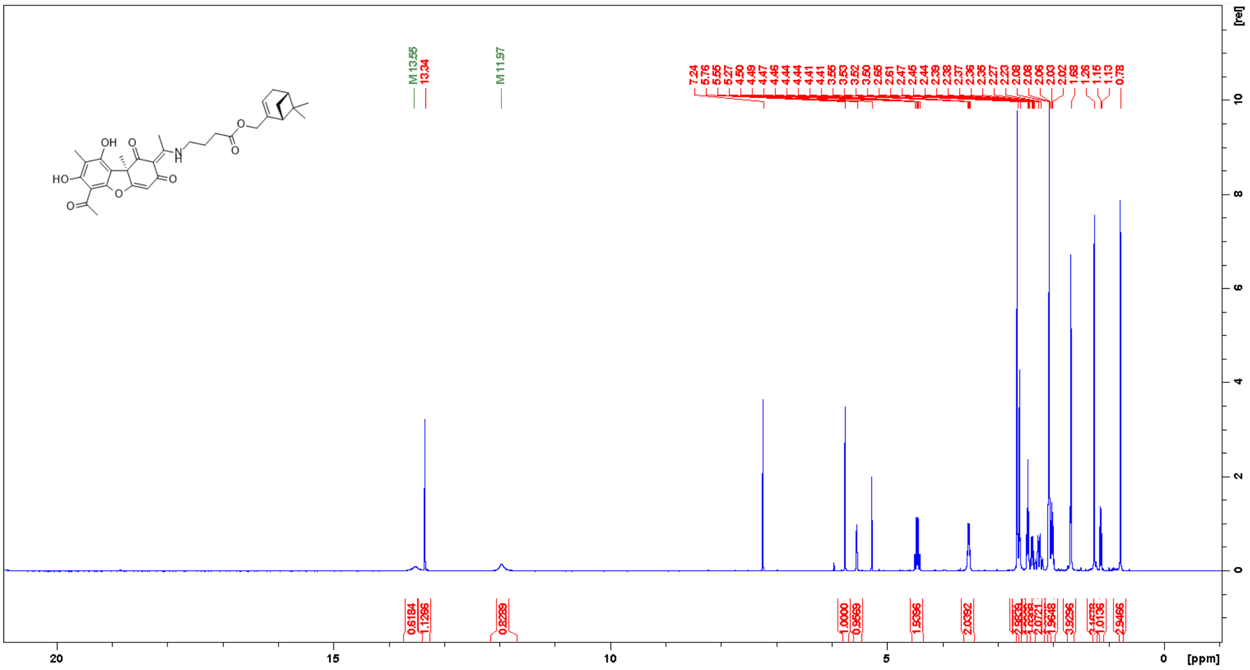

Figure S17. NMR ^{1}H spectra of **13d.**

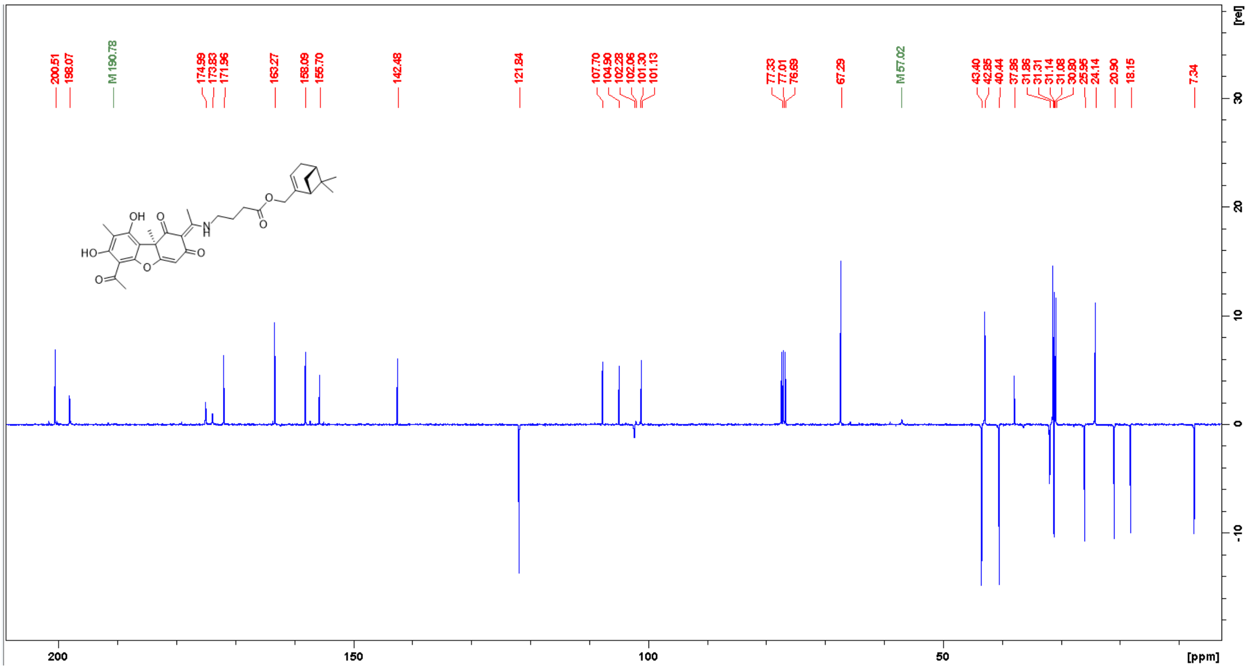

Figure S18. NMR ^{13}C (J-MOD) spectra of **13d.**

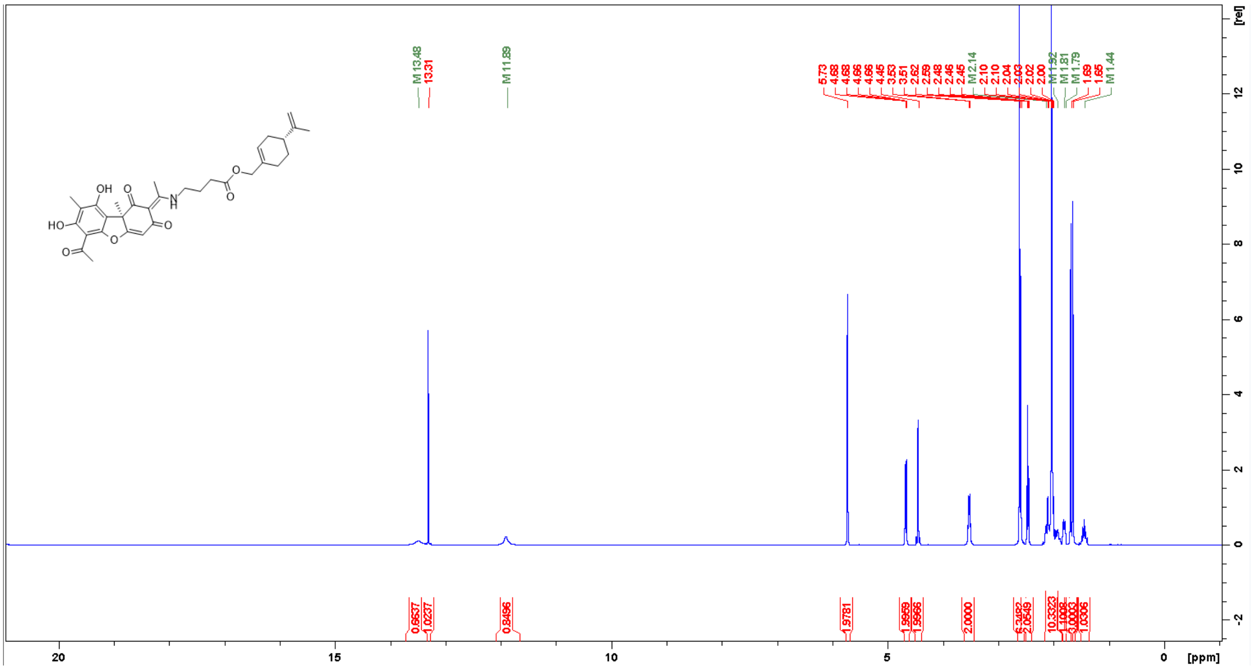

Figure S19. NMR ^1H spectra of **13e.**

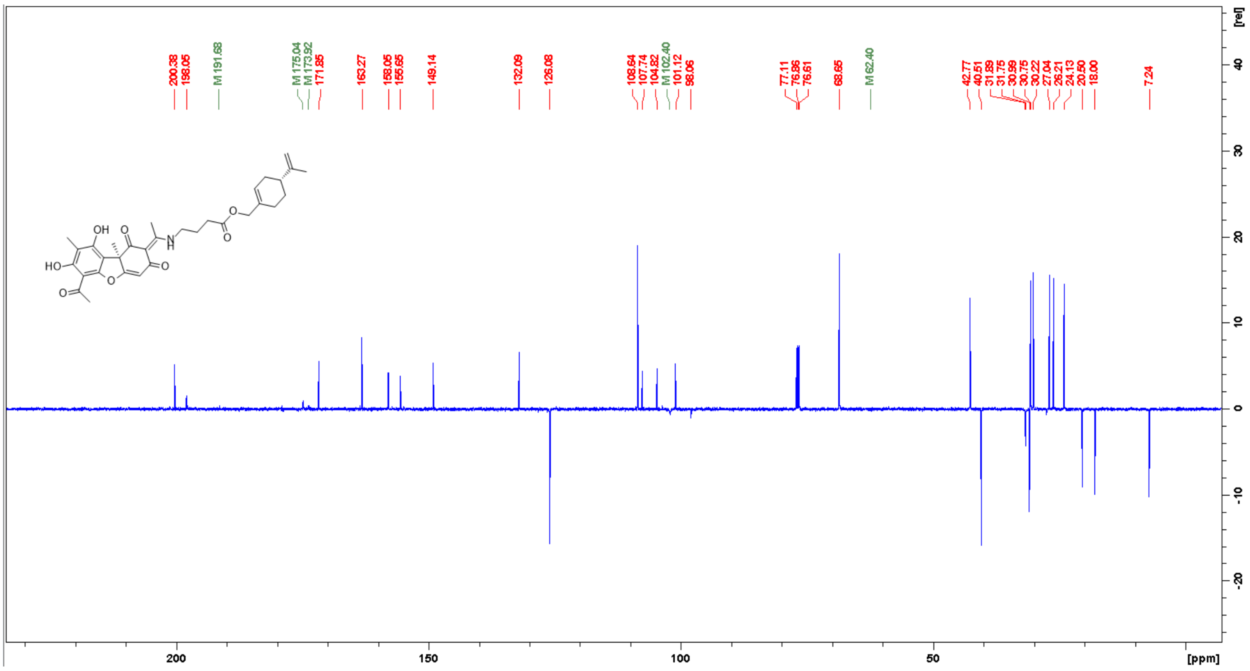

Figure S20. NMR ^{13}C (J-MOD) spectra of **13e.**

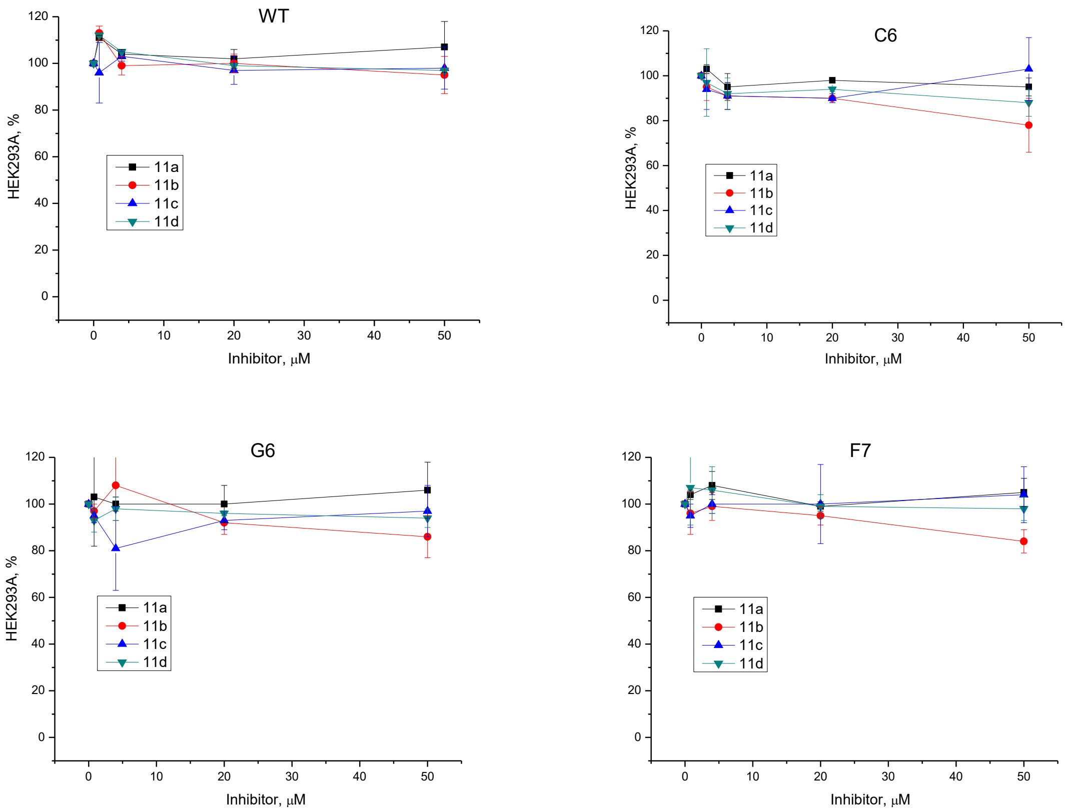

Figure S21. TDP1 inhibitors' intrinsic cytotoxicity on HEK293A WT and TDP1 -/- cells (C6, G6, F7), dose-dependent action of the derivatives **11a, 11b, 11c, 11d**.

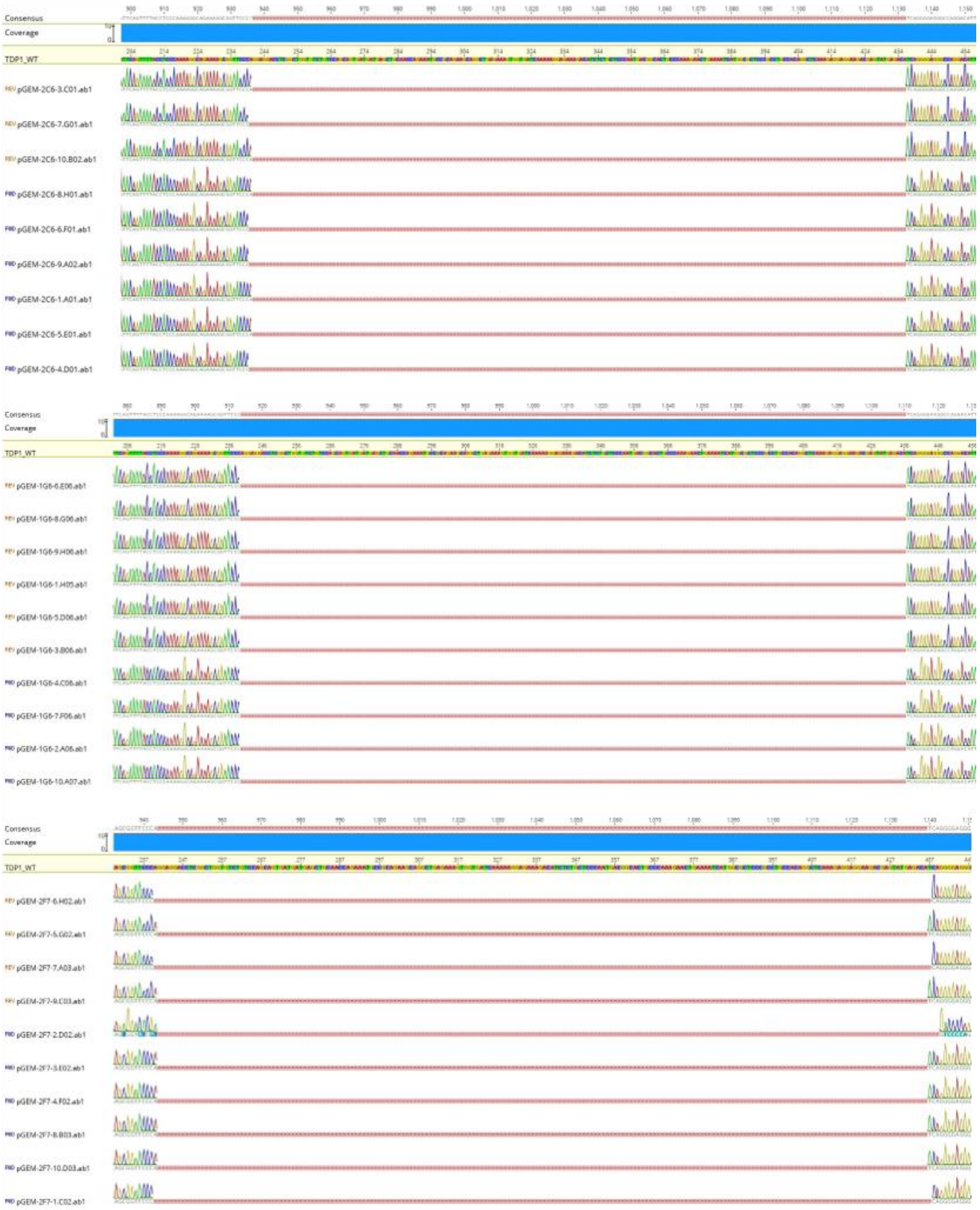

Figure S22. Verification of CRISPR/Cas9-mediated deletions in the TDP1 gene by genome DNA sequencing. Alignment of plasmid clones sequenograms (C6, G6, F7) with the wild-type sequence of the TDP1 gene revealed the presence of deletions that shift the reading frame and potentially disrupt the synthesis of the corresponding protein.

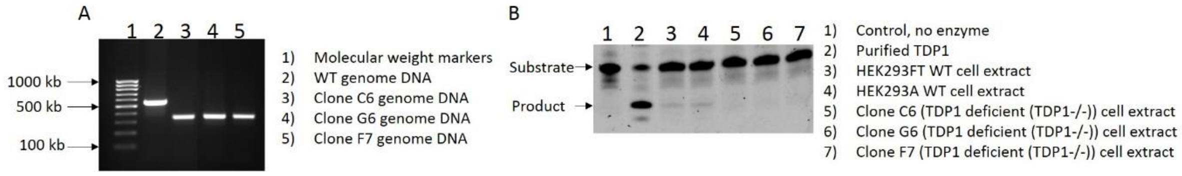

Figure S23. Analysis of the HEK293 TDP1 -/- clones. A. PCR analysis for the presence of CRISPR/Cas9-mediated deletions in TDP1 gene. PCR analysis showed homozygous deletions in the first protein coding exon (third exon in mRNA, NM_001008744.2) of the TDP1 gene in clones C6, G6, F7 (lanes 3-5). B. Identification of TDP1 3′-phosphotyrosyl cleavage activity in the HEK293 cell extracts: HEK293FT WT (lane 3), HEK293A WT (lane 4) and clones C6, G6, F7 cells (lanes 5-7). There was no established cleavage activity in the clones C6, G6, F7 cell extracts in contrast to control WT cell extracts and purified TDP1.

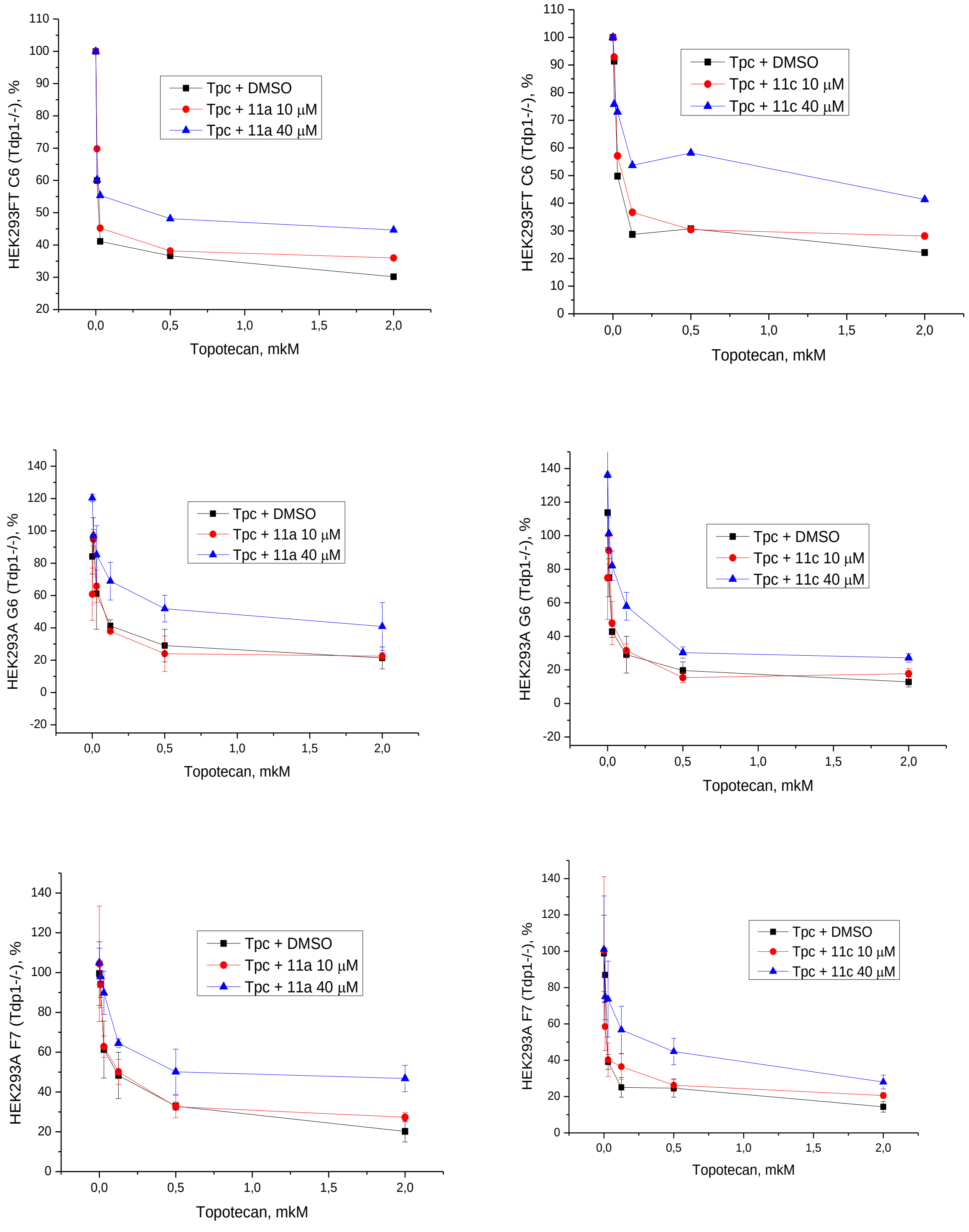

Figure S24. Topotecan cytotoxicity on HEK293A TDP1 -/- (clones C6, G6, F7) cells, dose-dependent action of topotecan in combination with **11a** or **11c** compounds by colorimetric test.

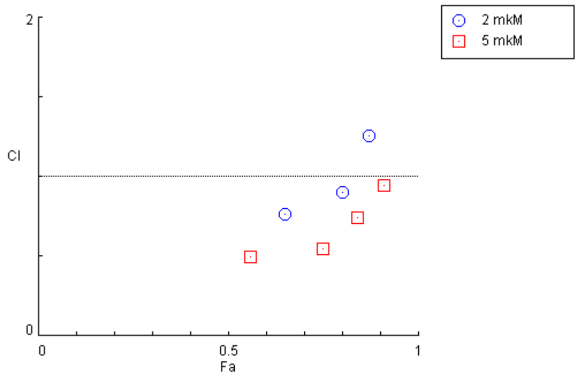

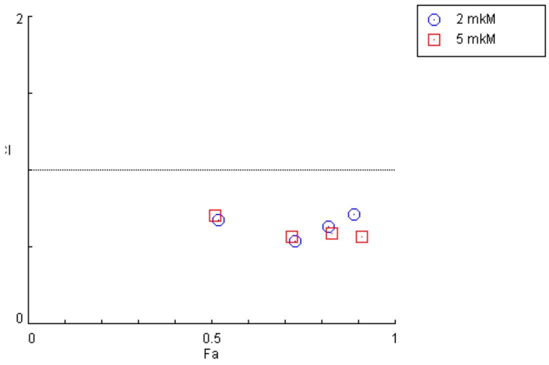

Figure S25. Topotecan cytotoxicity on HeLa cells, dose-dependent action of topotecan in combination with **11a** (left) or **11c** (right) compounds by colorimetric test and CompuSyn version 1.0 software.

Table S1. The binding affinities as predicted by the scoring functions used to the catalytic binding site.

Ligand	ASP	CS	ChemPLP	GS	**IC_{50} μM**
**13d**	42.4	31.4	72.7	64.5	>10
**13b**	39.0	30.6	76.5	66.5	>10
**11a**	36.3	30.2	68.7	62.9	0.4
**11c**	36.6	33.3	66.3	58.2	0.32
**11e**	33.8	31.7	62.9	59.2	>10
**11d**	37.9	31.7	68.6	62.7	0.23
**11b**	37.5	32.5	71.6	63.7	0.32
**13a**	38.8	31.5	77.1	64.5	>10
**13c**	40.2	32.4	75.5	64.7	>10
**13e**	42.9	33.4	74.9	64.3	>10
**R^2**	0.786	0.166	0.000	0.001	
Active Average:	37.1	31.9	68.8	61.9	
Inact. Average:	39.5	31.8	73.3	63.9	
TG7 co-cryst.	35.5	34.0	66.9	63.3	
RMSD (Å)	6.377	5.255	4.438	4.723	

To validate the robustness of the docking protocol the predicted poses were overlain with the co-crystallized ligand and the root-mean-square deviation (RMSD) was calculated for the heavy atoms. Relatively poor predictions were obtained in the 4.4 – 5.2 Å range for the scoring functions (see Table S1); the two carboxylic groups, on the phenyl ring, form ionic bonds with Lys265 and Lys495, which is correctly predicted by GOLD, but it places the bicyclic heteroring in a different position than the co-crystallized conformation. This resulted in the poor RMSD values; different docking locations, settings and presence of crystalline water molecules were tried but did not improve the overlap. The binding of the TG7 ligand is dominated by the ionic bonds, which are quite strong, and it is relatively unusual for two ionic bonds to drive the binding interaction making difficult for the algorithm to arrive at a similar conformation as the X-ray structure.

The binding predictions, for the catalytic pocket occupied by TG7, of the four scoring functions used are given in Table S1; all the ligands show reasonable scores. A good correlation for ASP is seen (R^2 – 0.786) with the measured IC_{50} values but not for the other scoring functions. When the averages of the scores of the ligands with IC_{50} values >10 μM were calculated and compared with their active counterparts no significant difference was seen (Table S1).

Table S2. The binding affinities as predicted by the scoring functions used to the allosteric binding site.

Ligand	ASP	CS	ChemPLP	GS	**IC_{50} μM**
**13d**	43.2	28.6	72.7	63.06	>10
**13b**	47.56	27.9	82.16	63.83	>10
**11a**	39.68	25.7	64.53	57.17	0.4
**11c**	40.39	24.9	64.26	56.07	0.32
**11e**	37.53	24.9	63.88	51.44	>10
**11d**	36.48	24.5	59.33	54.94	0.23
**11b**	40.12	27.2	68.85	59.08	0.32
**13a**	46.14	27.8	74.85	61.25	>10
**13c**	42.94	31.9	75.63	65.56	>10
**13e**	46.82	31.0	80.85	57.5	>10
**R^2**	0.559	0.196	0.329	0.288	
Active Average:	39.2	25.6	64.2	56.8	
Inacti. Average:	44.0	28.7	75.0	61.0	

Table S3. The molecular descriptors and their corresponding Known Drug Indexes 2a and 2b ($KDI_{2a/2b}$). The R^2 numbers derived do not contain the IC_{50} > 10 μM values.

Ligand	**RB**	**MW** g/mol	**HD**	**HA**	**Log P**	**PSA** Å	**2A**	**2B**	**IC_{50} μM**
**13d**	11	563.6	1	9	5.2	160.1	3.89	0.06	>10
**13b**	14	565.7	1	9	5.7	161.7	3.50	0.02	>10
**11a**	11	481.6	1	7	5.2	125.2	4.68	0.19	0.4
**11c**	8	491.6	1	7	4.9	125.0	5.06	0.35	0.32
**11e**	8	477.6	1	7	4.7	123.8	5.14	0.39	>10
**11d**	7	477.6	1	7	4.5	124.9	5.27	0.46	0.23
**11b**	10	479.6	1	7	5.1	125.4	4.82	0.24	0.32
**13a**	15	567.7	1	9	5.8	161.6	3.43	0.01	>10
**13c**	12	577.7	1	9	5.5	161.4	3.66	0.04	>10
**13e**	12	563.6	1	9	5.4	160.0	3.75	0.04	>10
**R^2**	0.799	0.999*	0.000	0.000	0.849	0.332	0.859	0.861	
Active Average:	9	482.6	1	7	4.9	125.1	4.96	0.31	
Inactive Average:	12	552.6	1	8.67	5.4	154.8	3.89	0.09	
Difference:	3	70.1	0	1.67	0.5	29.6	-1.06	-0.22	

*OL10-120 is an outlier and not included in the R^2.

Table S4. Definition of lead-like, drug-like and Known Drug Space (KDS) in terms of molecular descriptors. The values given are the maxima for each descriptor for the volumes of chemical space used.

	**Lead-like Space**	**Drug-like Space**	**Known Drug Space**
Molecular weight (g mol^{-1})	300	500	800
Lipophilicity (Log P)	3	5	6.5
Hydrogen bond donors (HD)	3	5	7
Hydrogen bond acceptors (HA)	3	10	15
Polar surface area ($Å^2$) (PSA)	60	140	180
Rotatable bonds (RB)	3	10	17

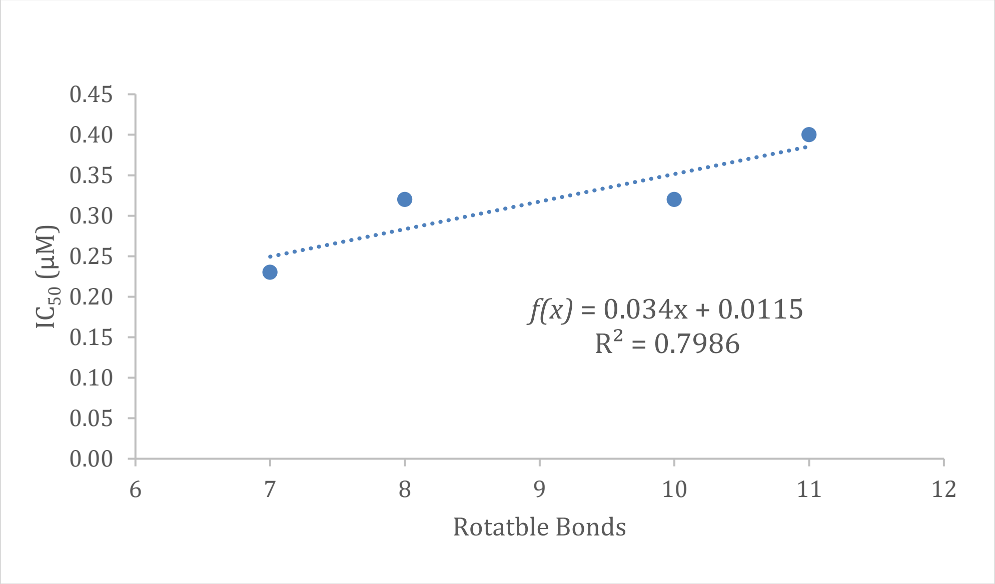

Figure S26. The correlation of the IC_{50} values of the active ligands with RB.

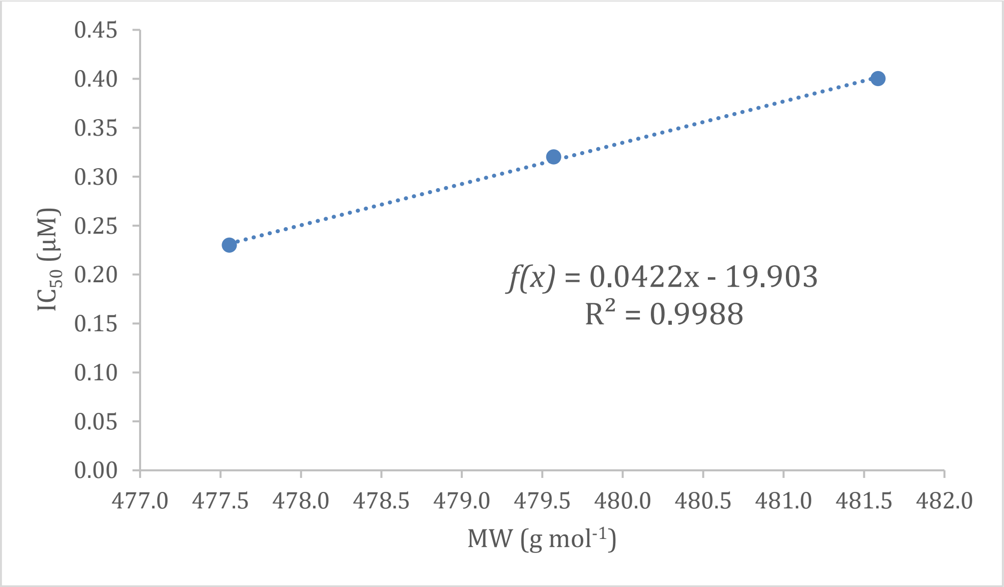

Figure S27. The correlation of the IC_{50} values of the active ligands with MW. **11c** is an outlier and not included in the R^2.

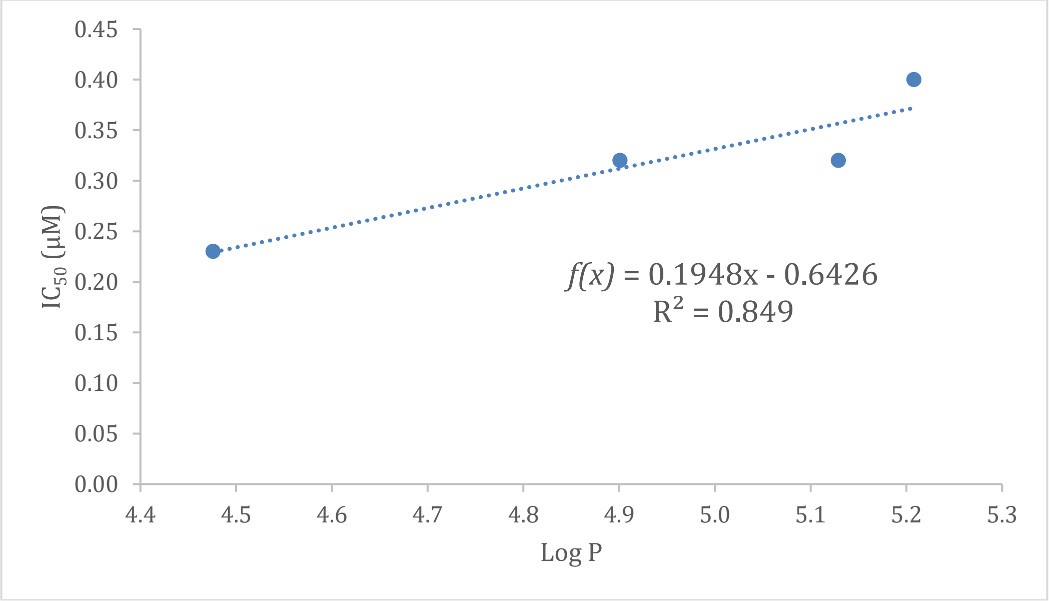

Figure S28. The correlation of the IC_{50} values of the active ligands with Log P.

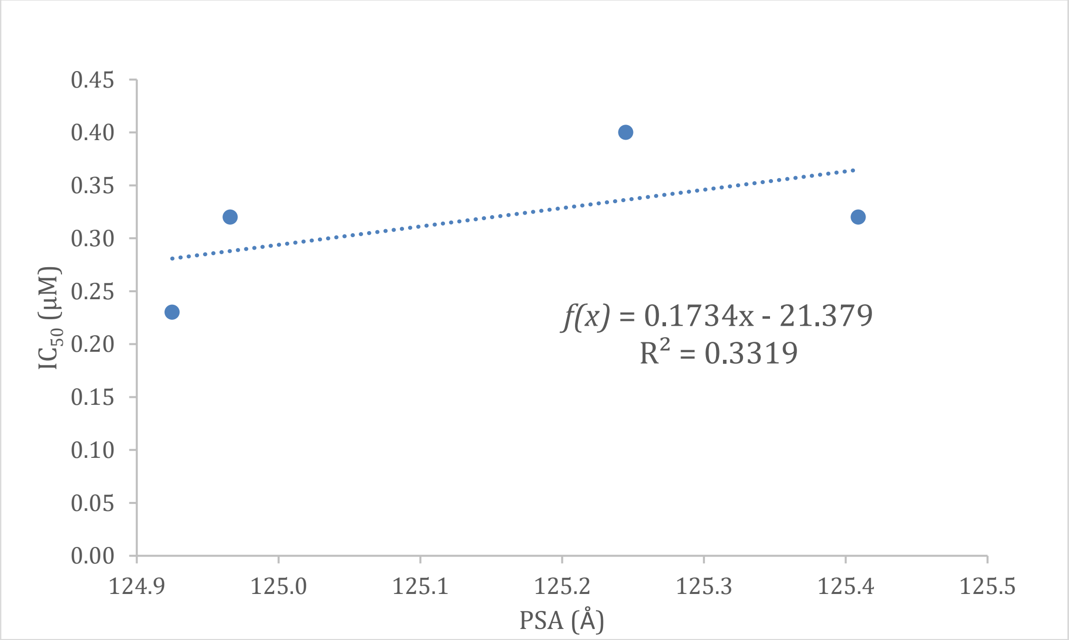

Figure S29. The correlation of the IC_{50} values of the active ligands with PSA.

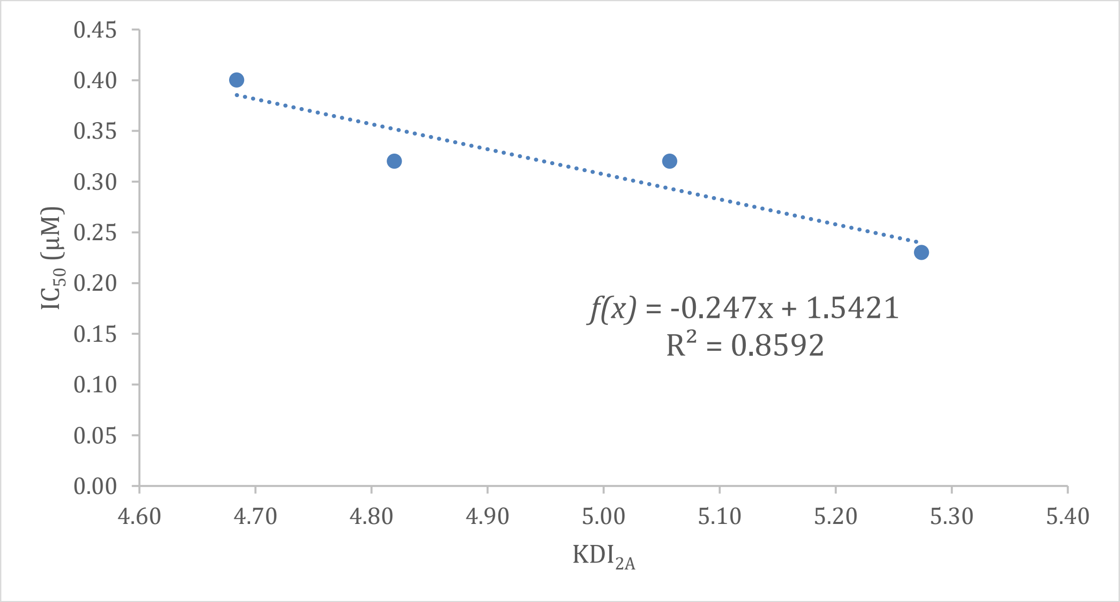

Figure S30. The correlation of the IC_{50} values of the active ligands with KDI_{2A}.

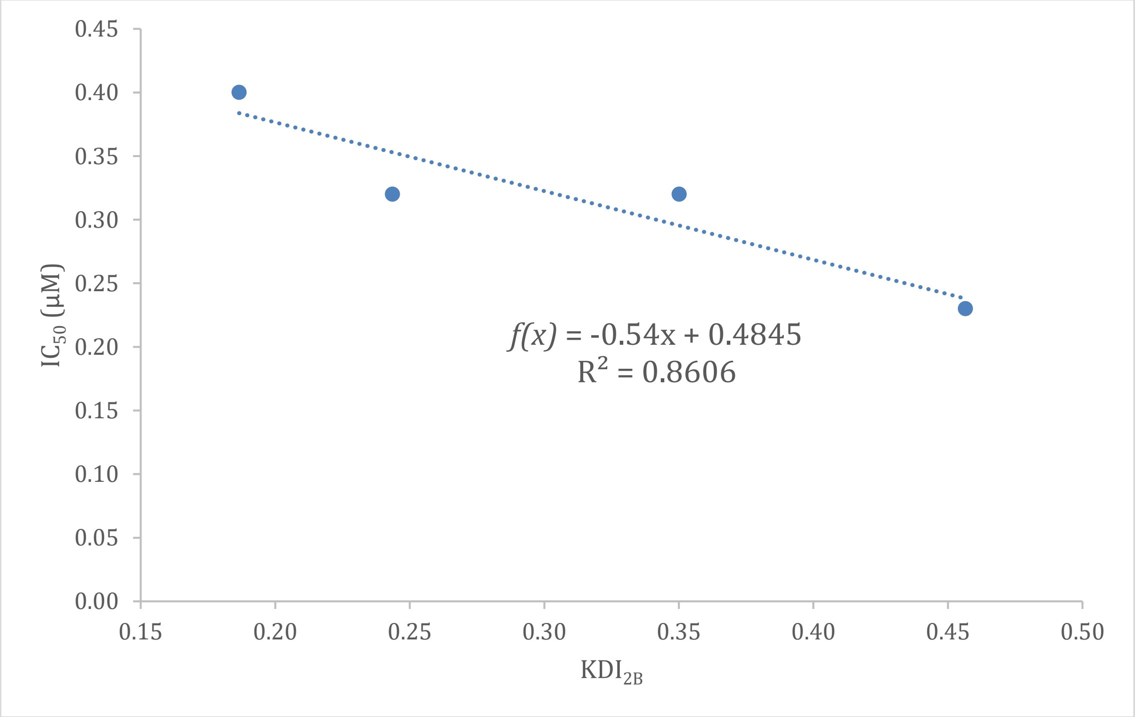

Figure S31. The correlation of the IC_{50} values of the active ligands with KDI_{2B}.
